# Highly efficient *in vitro* and *in vivo* delivery of functional RNAs using new versatile MS2-chimeric retrovirus-like particles

**DOI:** 10.1038/mtm.2015.39

**Published:** 2015-10-21

**Authors:** Anne Prel, Vincent Caval, Régis Gayon, Philippe Ravassard, Christine Duthoit, Emmanuel Payen, Leila Maouche-Chretien, Alison Creneguy, Tuan Huy Nguyen, Nicolas Martin, Eric Piver, Raphaël Sevrain, Lucille Lamouroux, Philippe Leboulch, Frédéric Deschaseaux, Pascale Bouillé, Luc Sensébé, Jean-Christophe Pagès

**Affiliations:** 1 Université François Rabelais de Tours, INSERM UMR 966, Tours, France; 2 UMR UPS/CNRS 5273, EFS-PM, INSERM U1031, Toulouse, France; 3 Vectalys, Bâtiment Canal Biotech 2, Parc Technologique du Canal 3, Toulouse, France; 4 Institut du Cerveau et de la Moelle (ICM), Université Pierre et Marie Curie, CNRS UMR7225; INSERM U1127, Biotechnologies and Biothérapies Team, Paris, France; 5 CEA/Université Paris Sud (UMR-E 007), Institut of Emerging Diseases and Innovative Therapies (iMETI), CEA de Fontenay aux Roses, Fontenay aux Roses, France; 6 INSERM UMRS 1064, Centre Hospitalier Universitaire (CHU) Hôtel Dieu, Nantes, France; 7 Institut de Transplantation Urologie Néphrologie (ITUN), Université de Nantes, Nantes, France; 8 CHRU de Tours, Laboratoire de biochimie et biologie moléculaire, Tours, France

## Abstract

RNA delivery is an attractive strategy to achieve transient gene expression in research projects and in cell- or gene-based therapies. Despite significant efforts investigating vector-directed RNA transfer, there is still a requirement for better efficiency of delivery to primary cells and *in vivo*. Retroviral platforms drive RNA delivery, yet retrovirus RNA-packaging constraints limit gene transfer to two genome-molecules per viral particle. To improve retroviral transfer, we designed a dimerization-independent MS2-driven RNA packaging system using MS2-Coat-retrovirus chimeras. The engineered chimeric particles promoted effective packaging of several types of RNAs and enabled efficient transfer of biologically active RNAs in various cell types, including human CD34^+^ and iPS cells. Systemic injection of high-titer particles led to gene expression in mouse liver and transferring Cre-recombinase mRNA in muscle permitted widespread editing at the ROSA26 locus. We could further show that the VLPs were able to activate an osteoblast differentiation pathway by delivering *RUNX2-* or *DLX5*-mRNA into primary human bone-marrow mesenchymal-stem cells. Thus, the novel chimeric MS2-lentiviral particles are a versatile tool for a wide range of applications including cellular-programming or genome-editing.

## Introduction

Achieving curative gene therapy for genetic diseases has been a remarkable reward after 30 years of bench-to-bedside transfer.^1^ We now foresee the advent of a medical revolution with cellular reprogramming and genome editing, alone or in combination.^1^ In the cell and gene therapy era, the need for improved targeted expression of cellular factors is critical.^2,3^ Moreover, for biological and safety reasons, the lifelong persistence of foreign genetic information is not desirable in many cases.^4^ Protein delivery has recently allowed transient expression in various cell types.^5,6^ RNA transfer also remains relevant and has been extensively developed with chemical, viral, and biophysical techniques. Importantly, RNA transfer allows transient expression and tuneable control.^7^

By exploiting the mRNA nature of the retroviral genome,^8^ Baum and others converted murine oncoretroviruses into RNA transfer vectors,^9–11^ which was then extended to other retroviruses.^12,13^ mRNA delivery from retroviral vectors involved the natural Ψ sequence and knockdown of molecular determinants for reverse transcription, or modification of cellular mRNA by adding the retroviral packaging sequence.^10,14^ Hence, these platforms feature viral constraints in genome recruitment and the use of the canonical viral-packaging sequence restrains delivery to no more than two RNAs per particle.^8^ An alternative packaging system not relying on the dimerization of the genomic RNA could improve the efficiency of transduction.

To improve RNA transfer by retroviral platforms, we designed novel chimeric particles that rely on the well-characterized interaction between bacteriophage MS2-Coat protein and the MS2-RNA genome.^15^ Through the physiological recognition of its cognate RNA, MS2-Coat governs the replication-to-assembly transition during the *leviviridae* lytic cycle.^16^ This interaction involves a 19-nt stem-loop structure within the phage replicase gene.^17^ MS2-Coat protein systems have wide application for studying RNA biology and fluorescent MS2-Coat-protein have been used to track cellular RNAs.^18,19^ Recently, MS2-Coat was used for semisynthetic gene delivery.^20,21^ Here, we challenged retroviral plasticity to tolerate insertion of an MS2-Coat domain allowing for heterogeneous RNA recruitment.^6^ Several retroviral sequences were amenable to modification in retroviral-based vectors.^6,22^ In this study, we evaluated the capacity to transfer MS2-Coat-binding RNAs from our redesigned HIV-1-derived vectors. Having shown particle formation, and confirmed by quantitative assays that MS2 chimeric RNA lentiviral particles (MS2RLPs) contained significantly more than two RNAs, we achieved visible efficient transfer of functional RNAs *in vitro*, with no need for selection. Concentrated chimeric particles led to significant liver and muscle transduction *in vivo*. Finally, lentiviral cargoes containing mRNAs coding osteogenic transcription factors triggered a shift in the transcription program of primary bone-marrow mesenchymal stem cells (MSCs).

## Results

### Chimeric MS2-Coat-lentiviral particles promote efficient packaging of MS2-RNA

**Figure 1:**
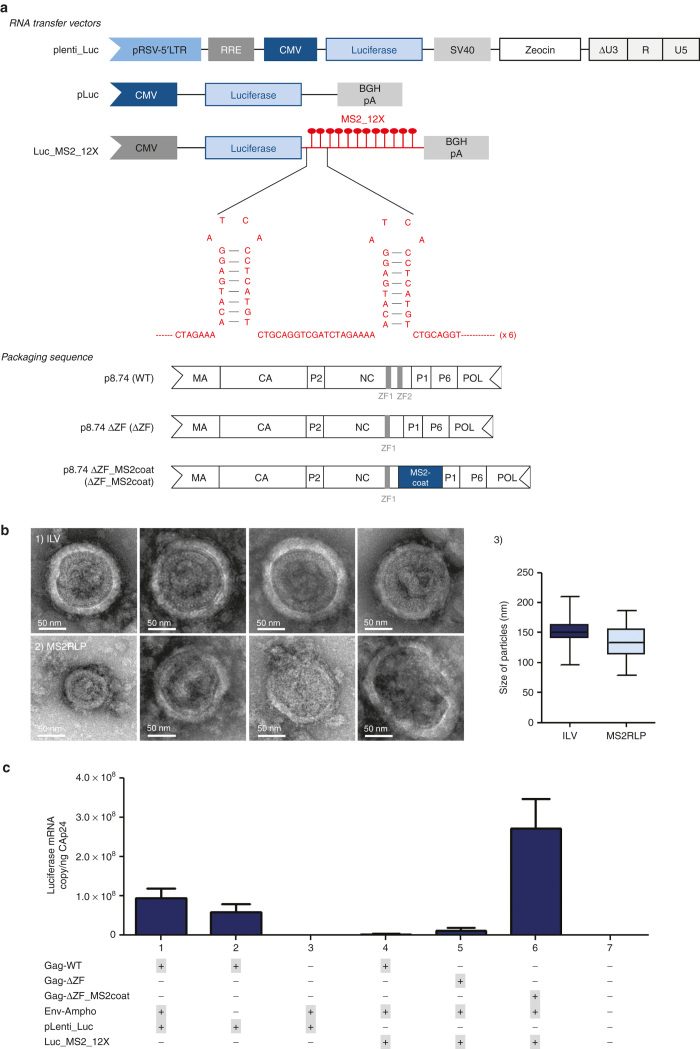
MS2-driven RNA packaging into chimeric MS2-coat lentiviral particles. (**a**) Vectors for mRNA recruitment into MS2RLP. RNA transfer vector: control and reporter constructs. plenti_Luc: lentiviral vector serving as positive control for Ψ-dependent packaging. pLuc: a pcDNA-derived vector expressing the luciferase gene. Luc_MS2_12X: model construct designed to evaluate MS2-Coat protein based recruitment, the luciferase gene expressed under the CMV promoter contains 12 copies of the 19-nt RNA stem-loop from MS2 bacteriophage (MS2_12X). BGH-pA: Bovine growth hormone poly-adenylation signal. In red, detail of the sequence for two repeats. Packaging sequence: p8.74 (WT): standard HIV-1 trans-complementation, p8.74 ΔZF (ΔZF): derived from WT by deletion of the ZF2 in Nucleocapsid (NC). p8.74 ΔZF-MS2coat (ΔZF_MS2coat): vector containing MS2-Coat protein in place of the ZF2. CA, capsid; MA, matrix; MS2-coat, coat protein from MS2 bacteriophage; NC, nucleocapsid; POL, polymerase. (**b**) Transmission electron microscopy. (1) Control integrative lentiviral vectors; (2) MS2RLPs, picture include the smaller and larger particles. Both particles were observed by transmission electron microscopy in negative staining conditions using a LaB6 JEOL JEM2100 electron microscope (magnification: ×52,800). Bars 50 nm. (3) Boxplot, distribution of the average size of particles measured from 52 pictures of integrative-lentiviral (ILV) (dark blue) and 35 of MS2RLP (light blue). Images and counts were obtained from a single production of each vector. (**c**) RT-qPCR analysis of luciferase mRNA level in MS2RLPs. Control and MS2RLP samples of DNAse-treated supernatant collected 48 hours after transfection. In all particles collected, no DNA amplification was observed without an RT step (data not shown). The analyzed volume was defined after normalization to CAp24 concentration in the supernatant. Data are mean ± SD (*n* = 3, in duplicate). Gag-WT: lentiviral vector expressing a wild-type Gag. Gag-ΔZF: lentiviral vector expressing a ZF2 deleted Gag. Gag-ΔZF_MS2Coat: lentiviral vector expressing Gag with ZF2 replaced by MS2coat sequence. Env-Ampho: amphotropic envelope. pLenti_Luc: lentiviral vector expressing the Luciferase gene. Luc_MS2_12X: mRNA containing 12 repetitions of MS2 RNA stem loop and the Luciferase coding sequence. Combination of Gag-ΔZF_MS2Coat, Luc_MS2_12X.

To date, because the natural mode of packaging implies RNA dimerization within Ψ, the number of RNAs within each particle is limited to 2.^8,23^ To increase retroviral mRNA delivery, we engineered dimerization-independent RNA recruitment by exploiting the specific interaction between the phage MS2-Coat protein and MS2 RNA genome in a lentiviral context. For this, we needed to design two components, the biologically active RNA to be recruited and the recruiting particle. The minimal 19-nt stem-loop sequence required for interaction between the phage MS2-Coat protein and MS2 RNA was introduced in multiple copies^24,25^ into a eukaryotic expression vector. We first cloned the stem-loop at the 3’ end of a reporter gene, luciferase (Luc_MS2_12X) (Figure 1a). Next, to obtain the packaging of MS2-stem-loop containing RNA into a particle, we engineered an HIV-1-based complementation vector.^26^ HIV-1 recruits its genomic RNA via a complex process,^8,23^ in which the viral nucleocapsid protein (NC) has a central role. Because NC has several other functions in HIV-1 biology, we could not simply replace it (for review, see ref. 27). HIV-1 NC contains two zinc finger domains (ZF1 and ZF2) involved in RNA interaction; ZF2 was shown to be dispensable for particle budding^22^ and was thus selected for modification. We first generated a reference and negative control construct (p8.74 ΔZF, Figure 1a) and next introduced in-frame the sequence encoding bacteriophage MS2-Coat protein, replacing ZF2 (p8.74 ΔZF_MS2-coat) (Figure 1a).

To determine whether this system could allow MS2-lentiviral particle (MS2RLPs) formation and mRNA recruitment, the Luc_MS2_12X vector was transfected along with each HIV-1 packaging construct (p8.74, p8.74 ΔZF, or p8.74 ΔZF_MS2coat). In the first experiments, MS2RLPs were pseudotyped by the 4070A amphotropic envelope, preventing unspecific release of cellular vesicles formed by the pantropic VSV-G.^28^

Measurements of the concentration of CAp24 in the supernatant from control and MS2RLPs transfected cells by enzyme-linked immunosorbent assay (ELISA) suggested that the packaging constructs supported particle formation (data not shown). This was confirmed by electron microscopy showing particles in the medium from transfected cells (Figure 1b). Morphologically, the majority of MS2RLPs appeared similar to conventional lentiviral vectors with only a slight change in shape and size (Figure 1b). Of note, these differences were associated with the presence of unprocessed Gag precursor in MS2RLPs (Supplementary Figure S1a).

Next, we analyzed uptake of luciferase-mRNA into the MS2RLPs. Quantitative reverse transcription polymerase chain reaction (RT-qPCR) confirmed the specific recruitment of Luc_MS2_12X mRNA into MS2RLPs (Figure 1c, 6 compared to 4 and 5). The parental construct allowed for packaging the Ψ-containing genome but it failed to recruit Luc_MS2_12X mRNA (Figure 1c, 1–2 compared to 4). Similarly, the p8.74 ΔZF control vector failed to package Luc_MS2_12X mRNA (Figure 1c, sample 5), which emphasizes the crucial role of the interaction between MS2-Coat protein and the MS2 19-nt stem-loop. Importantly, because the amount of luciferase RT-qPCR mRNA was normalized to that of particle load measured by CAp24, the higher signal obtained for MS2RLP-Luc suggested that MS2RLPs packaged more RNA molecules per particle than the wild-type Ψ-dependent lentiviral system (Figure 1c, compare sample 1 and 6). Since MS2RLPs had a shape close to that of regular lentiviral vectors (Figure 1b), we hypothesized that the CAp24 concentration was proportional to the number of particles released both for the standard lentiviral vectors and for MS2RLP. This was confirmed by a western blot analysis of MS2RLP and integrative-lentiviral (ILV) particles showing that both particles contained detectable CAp24, while MS2RLP had a higher proportion of Gag precursor (Supplementary Figure S1a). To accurately estimate the MS2RLP RNA contents, we designed a dilution-based method (see Materials and Methods and Supplementary Figure S1b). These results allowed us to show that MS2RLP contained around 2.8 times more RNA than a standard vector (Supplementary Figure S1b). Considering that Ψ-dependent vectors contained two viral genomes, we estimated the number of RNA molecules per MS2RLP to be around 5 to 6 (Figure 1c and Supplementary Figure S1b).

### Kinetic of MS2RLP-delivered RNA expression

**Figure 2:**
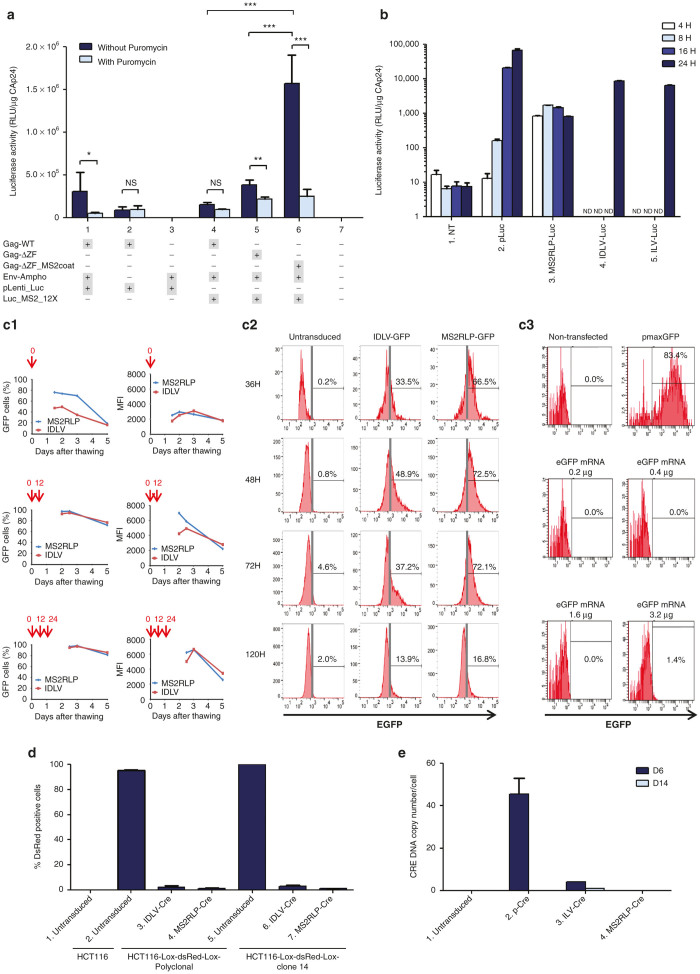
MS2RLP allow efficient RNA expression following mRNA transfer. (**a**) Luciferase activity after MS2RLP transfer of luciferase-MS2 mRNA into 293T cells. 293T cells without (dark blue) or pretreated with 3 µg/ml puromycin (light blue) were transduced with 100 µl crude supernatant from MS2RLPs containing luciferase mRNA (MS2RLP-Luc) and controls harvested 48 hours post-transfection. At 3 hours post-transduction, cells were extensively washed, lysed, and luciferase activity was measured. Plotted results were obtained after normalization to CAp24 antigen except for sample 3 and 7 for which a representative extraction aliquot was studied. NS, not significant; (* < 0.05); (** < 0.01), and (*** < 0.001) by Wilcoxon-Mann-Whitney test. (**b**) Kinetics of luciferase activity upon MS2RLP transfer into HCT116 cells. HCT116 cells were transduced with MS2RLP-Luc, integrase-deficient lentiviral vector expressing luciferase (IDLV-Luc); 20 pg of CAp24/cell in both cases. Controls were an integrative lentiviral vector expressing luciferase (ILV-Luc), 0.57 pg CAp24/cell and the transfection of a recombinant luciferase expression plasmid (pLuc). Cells were washed and lysed, and luciferase activity was measured at various times post-transduction or transfection (see legend). NT, nontransfected or nontransduced cells. (**c1** and **c2**) Green fluorescent protein (GFP) expression into hematopoietic cells. CD34^+^ cells were thawed, suspended in culture medium at a concentration of 1.5 million cells per ml, and plated in 96-well plates. 200,000 cells were transduced with no vector, 13 µl of concentrated IDLV-GFP or 13 µl of concentrated MS2RLP-GFP, in the presence of protamine sulfate (8 µg/ml). Cells were transduced once, (right after thawing, top panels), twice (after thawing and 12 hours later, middle panels), or three times (after thawing, 12 and 24 hours later, bottom panels). GFP-expressing cells and median fluorescence intensities (MFI) were analyzed by flow cytometry, 36 hours after the last transduction and beyond (2c1). Detailed results are given for the one-shot transduction experiment, as measured 36, 48, 72, and 120 hours after transduction (2c2). (**c3**) 50,000 CD34^+^ cells were transfected either with 0.4 µg of a GFP expressing plasmid (pmaxGFP) or with a GFP mRNA using the 4D-nucleofector X unit 1 day after cell thawing. GFP cell expression was analyzed 2 days later by Flow cytometry (BD FACSCanto II, Becton Dickinson) and the Diva software (Becton Dickinson). (**d**) MS2RLPs transfer of Cre recombinase mRNA into HCT116-Lox-dsRed-Lox cells. Polyclonal or clonal HCT116-Lox-dsRed-Lox cells were transduced with 20 pg CAp24/cell of a IDLV-Cre (samples 3 and 6) or MS2RLP containing Cre recombinase mRNA (MS2RLP-Cre) (samples 4 and 7). At 17 days after transduction, fluorescence was measured and quantified by FACS analysis. Sample 1: control normal HCT116 cells. Samples 2 and 5: Untransduced HCT-Lox-dsRed-Lox cells. Data are mean ± SD (*n* = 3). (**e**) Detection of Cre recombinase DNA sequence in transduced cells. HCT116-Lox-dsRed-Lox cells transduced with MS2RLP-Cre, ILV-Cre, or transfected by a plasmid expressing the Cre recombinase, were maintained in culture for 6 (dark blue) to 14 days (light blue). After DNA extraction, a quantitative PCR assay allowed for specific Cre recombinase gene detection.

**Figure 3:**
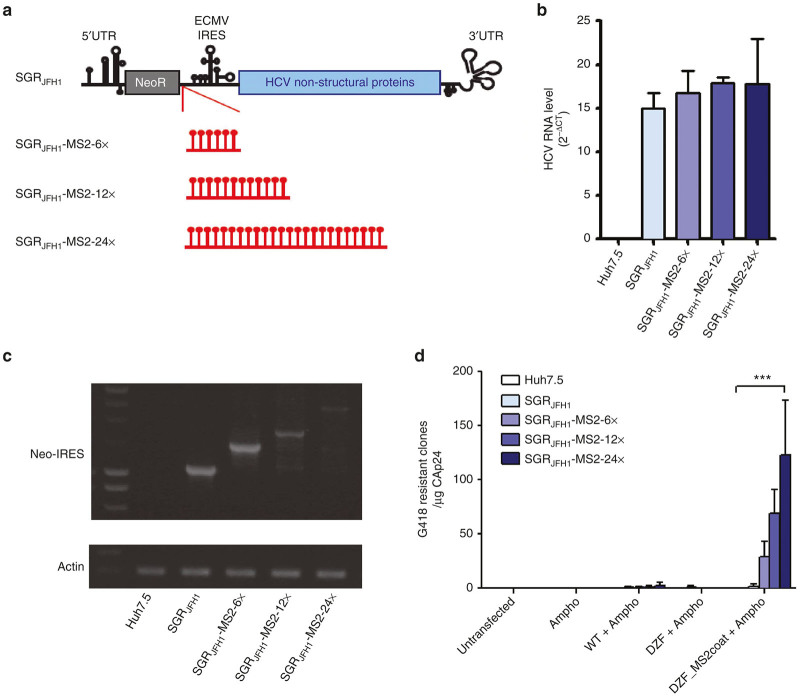
MS2RLP transfer of HCV subgenomic-replicon RNA. (**a**) Constructs for MS2RLP transfer of hepatitis C virus (HCV) subgenomic replicon. MS2-modified HCV SGR_JFH1_ replicons: MS2-tagged bicistronic HCV RNA was generated by insertion of 6×, 12×, or 24 × 19-nt MS2 stem-loop at a PmeI restriction sites of the SGR_JFH1_. (**b**) Replication activity of modified replicons. Constructs were transcribed *in vitro* and transfected into Huh7.5 naive cells. Histograms show RT-qPCR analysis of G418-treated resistant clones probed for HCV RNA replication efficiency. Control SGR_JFH1_, SGR_JFH1_-MS2-6X, SGR_JFH1_-MS2-12X, SGR_JFH1_-MS2-24X; light to dark blue bars respectively. (**c**) Integrity of MS2 stem-loop in replicons. Following selection, MS2 insertion integrity was confirmed by RT-PCR with primers amplifying a region comprising the different repeats. A cellular actin RT-PCR was used as a control of RNA integrity following extraction (lower panel). Neo-IRES: sequence containing the forward and reverse primers. (**d**) Mobilization of MS2-tagged HCV replicons by MS2RLP in transfected Huh7.5 cells. Huh7.5 expressing the modified replicons was transfected with an Amphotropic expression plasmid (Ampho, sample 2); the WT p8.74 packaging construct and the Amphotropic plasmids (WT+Ampho, sample 3); the ΔZF packaging and the Amphotropic plasmids (ΔZF+Ampho, sample 4) and the ΔZF_MS2coat and the Amphotropic plasmids (ΔZF_MS2coat+Ampho, sample 5). Filtered supernatants were used to transfect naïve Huh7.5 cells. After G418 treatment, resistant clones were fixed, crystal violet-stained and counted to quantify replicon mobilization. Mean ± SD (*n* = 4) analyzed by Kruskal-Wallis analysis (***P < 0.01).

We further showed that MS2RLPs drove rapid and efficient gene expression *in vitro*. To assess the functionality of the packaged MS2-12X mRNA, we assayed whether packaged RNAs enabled luciferase expression. Filtered supernatants, harvested 48 and 72 hours after transfection, were used to transduce 293T cells. Luciferase activity was measured 3 hours after transduction (Figure 2a, dark blue bars, samples 1–6). Luciferase expression was detected in cells transduced with MS2RLPs, but only at background levels in wild-type or ΔZF lentiviral control constructs (Figure 2a, dark blue bars; 4, 5 compared to 6), demonstrating that packaged mRNAs were functional. To ensure that the detected luciferase activity was due to mRNA transfer and subsequent translation, and not to pseudo-transduction, the passive transfer of luciferase protein, we pretreated recipient 293T cells with 3 µg/ml of the translation inhibitor puromycin at 1 hour before transduction (Figure 2a, light blue bars). Puromycin-treated cells showed only background luciferase activity as compared with nontreated cells (Figure 2a, sample 6, dark blue versus light blue bars). Therefore, luciferase activity measured in transfected cells resulted from the translation of the transferred mRNA. We next compared the kinetics of luciferase expression. MS2RLPs carrying the Luc mRNA (MS2RLP-Luc) were used to transduce HCT116 cells and luciferase expression was evaluated 4, 8, 16, and 24 hours later (Figure 2b). Control cells were transfected with a standard luciferase expression vector (pLuc) or transduced with integration-defective (IDLV-Luc) or integrative-lentiviral (ILV-Luc) vectors carrying the luciferase gene under the EF1 promoter. MS2RLP-driven luciferase expression was high and stable from 4 to 24 hours (Figures 2b and 3). A rapid decrease occurred in expression after 30 hours and was complete at 48 hours (Supplementary Figure S2a). Transfection of pLuc conferred detectable luciferase expression only after 8 hours and peaked at 24 hours, whereas IDLV or ILV conferred detectable luciferase expression only at 24 hours after transfer (Figure 2b; panels 2, 4, and 5). Therefore, the RNA delivered by MS2RLPs was more rapidly expressed than the plasmid-driven gene expression, conventional or nonintegrating lentiviral vectors. This showed that MS2RLPs allowed for rapid and high gene-expression.

We next evaluated MS2RLP functionality with a green fluorescent protein (GFP)-based reporter system using MS2-12X-GFP RNA (MS2RLP-GFP). For comparison, MS2RLP and GFP expressing IDLV particles were produced and concentrated side by side and identical volumes of vector supernatants were used to transduce human hematopoietic cord blood CD34^+^ cells at several time points after thawing (Figure 2c1). For cells transduced several times at 12 hours interval, more than 90% of the cells were GFP-positive cells with both vectors (Figure 2c1). Interestingly, the proportion of GFP-positive cells was twice higher with MS2RLP-GFP than with IDLV-GFP when cells were transduced immediately after thawing (Figure 2c1,2c2). This certainly reflected the need for CD34^+^ cells to be activated before efficient transduction.^29^ MS2RLP-GFP led to higher fluorescence intensity at early time points, but it tended to decrease slightly more rapidly than for IDLV-GFP (Figure 2c1).

In an effort to compare transduction and expression efficacy from MS2RLP-GFP to that obtained with other transfection methods, we used electroporation, one of the only methods capable of allowing efficient transfection of human primary CD34^+^ cells. For this, we used the 4D-Nucleofector technology and an appropriate kit from Lonza. In this condition, plasmid transfection gave a high rate of GFP-expressing cells with high fluorescence intensity (Figure 2c3). On the contrary, transfection of a stabilized EGFP mRNA (Trilink Biotechnologies) gave rise to almost undetectable level of expression (Figure 2c3). In order to confirm that the EGFP mRNA was functional, we transfected HEK293T cells with Lipofectamine 2000. More than 20% of the cells expressed the fluorescent protein at a high level (data not shown). This suggested that CD34^+^ cells were difficult to transfect using synthetic mRNA and the Nucleofector technology. We also tried to transfect the EGFP mRNA using the TransIT-mRNA transfection kit from Mirus Bio, but it failed to efficiently allow GFP expression in CD34^+^ cells (Supplementary Figure S2b). Altogether, these results suggested that MS2RLP allowed significant CD34^+^ hematopoietic cells transduction, even in the absence of cell activation by cytokines.

We further evaluated MS2RLP functionality of MS2-12X-tagged GFP RLP (MS2RLP-GFP) in cell lines. As for the luciferase, GFP was readily detectable, at high efficiency in more than 95% of Hela cells transduced with MS2RLP-GFP (Supplementary Figure S2c). However, we noticed distinct expression-kinetics, since GFP was detected only 24 hours following transduction and for up to 3 days (Supplementary Figure S2c). This difference could be due to the low sensitivity in GFP measurements by fluorescence-activated cell sorting (FACS), which would delay detection and to the longer half-life of the protein, which conferred prolonged detectability.^30^ As expected, the fluorescence decreased overtime and was cleared at 7 days post-transduction (Supplementary Figure S2c, lower right panel). Lastly, we showed that a single administration of MS2RLPs to human induced pluripotent stem cells (iPS) allowed for efficient GFP expression (Supplementary Figure S2d), and that MS2RLPs driven RNA-delivery resulted in a dose-dependent GFP signal (Supplementary Figure S2d).

### MS2RLPs allow efficient gene editing following Cre-mRNA transfer

These results prompted us to evaluate the expression of a protein active following its transfer into the nucleus of recipient cells. For this purpose, we cloned the Cre recombinase gene in a MS2-12X mRNA expression vector and evaluated its efficacy in promoting gene excision within cells stably expressing the dsRed protein from a floxable transgene (see methods). Following transfer, MS2RLP-Cre could induce the stable excision of the dsRed transgene (Figure 2d, sample 4 for the polyclonal population and sample 7 for clone 14). Of note the stability of excision suggested 100% efficacy following MS2RLP transfer. Equivalent excision was observed with an IDLV expressing Cre from an EF1 promoter (Figure 2d, sample 3 for the polyclonal population and sample 6 for clone 14). Remarkably, transfection of a Cre expression plasmid or stable expression of the Cre gene with an ILV, were less efficient in promoting excision (Supplementary Figure S2e). The fact that the target cells contained a mean of 6 floxable transgenes for the cell population and 13 floxable sites for the clone, implied that one single MS2RLP-Cre delivery was sufficient to excise several loci.

The RNAs packaged into MS2RLP do not contain any retroviral sequence involved in the reverse transcription process, which precluded their DNA conversion following transfer. We thus looked for a possible DNA integration of the Cre recombinase gene in transduced cells. As expected, no signal was detected for MS2RLPs and only ILV-Cre led to integrated copies (Figure 2e, sample 3 compared to 4). Taken together, our results validated the ability of the MS2RLP packaging system to promote efficient and specific mobilization of biologically active MS2-12X mRNA in all tested cell types.

### MS2RLPs deliver subgenomic viral RNAs

We next investigated the mobilization of other types of cytoplasmic RNA with MS2RLPs. Hepatitis C virus (HCV) replicon RNAs, that autonomously replicate by expressing nonstructural viral genes products were used as a model for uncapped RNA.^31^ To enable specific mobilization of HCV-replicon RNA, MS2 stem-loop repeats were inserted at the 3’ end of the neomycin selection marker coding sequence upstream the of EMCV IRES governing HCV nonstructural gene expression (Figure 3a).^31^ Resulting constructs SGR_JFH1_-MS2-6X, SGR_JFH1_-MS2-12X, and SGR_JFH1_-MS2-24X harboring 6, 12, and 24 copies of the MS2 motif respectively, were transcribed *in vitro* and transfected into Huh7.5 naive cells. Following G418 selection, we obtained resistant clones, except for the GND replication-defective HCV replicon (Supplementary Figure S3a). 5’UTR-specific RT-qPCR confirmed that G418-resistant Huh7.5 clones harbored HCV RNA and showed comparable replication efficiency (Figure 3b, samples 2–5). To check for MS2-loop loss during HCV replication, RT-PCR primers amplifying the MS2 insertion site were designed and confirmed the stability of the insert (Figure 3c). Replicon functionality was finally assessed by detection of NS3 and NS5A nonstructural proteins (Supplementary Figure S3b). Having confirmed that MS2-loop insertion was conserved during HCV replication, we next mobilized the modified replicon by using MS2RLPs. Replicon-harboring Huh7.5 cells were transfected with p8.74 ΔZF_MS2coat or controls and resulting particles were used to transduce naive Huh7.5 cells. No resistant clones were obtained from controls (Figure 3d, samples 2–4) or from wild-type SGR_JFH1_-replicating cells (Figure 3d, light blue bars compared to others in sample 5; Supplementary Figure S3). Conversely, neomycin-resistant clones were obtained from MS2RLPs produced in cells hosting an MS2-tagged HCV-replicon (Figure 3d, sample 5, for the three shades of blue). Interestingly, the mobilization efficiency appeared proportional to the number of inserted MS2 repeats (Figure 3d, sample 5). In transduced cells, specific HCV-RNA RT-qPCR analysis confirmed the efficient replication of HCV RNA (data not shown). As the size of the SGR_JFH1_-MS2-24X is approximately 10,000 nt, its recruitment within MS2RLPs suggested that the system could deliver long RNAs. Thus, MS2RLPs were efficient in mobilizing uncapped RNA with an exclusive cytoplasmic replication cycle, such as the HCV genome. Moreover, it indicated that MS2RLPs could be produced in Huh7.5.

### Efficient *in vivo* RNA delivery by MS2RLPs

We next determined whether MS2RLPs enabled systemic and localized *in vivo* expression in mice. The ability to deliver RNA *in vivo* would be valuable for several applications in research and the clinic. To ensure the use of purified particles at high titers, an important condition to obtain relevant results in *in vivo* applications, we used VSV-G pseudotypes (see methods). Among the possible routes for *in vivo* delivery, two representative modes of injection were evaluated.

**Figure 4:**
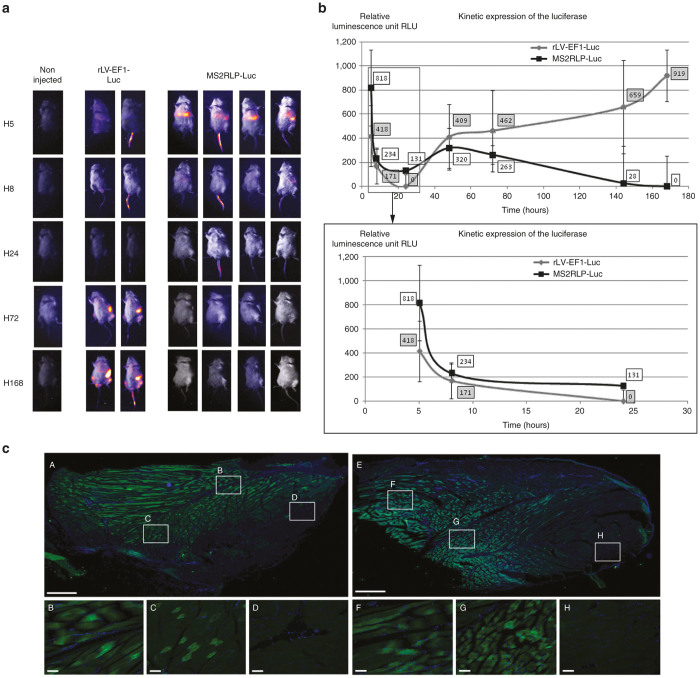
*In vivo* RNA delivery by MS2RLPs. (**a**) Bioluminescence *in vivo* analysis showing kinetics of luciferase expression in mice after injection of purified suspension of MS2RLP-Luc particles. A first group of animals received a suspension of the purified integrative lentiviral vector rLV-EF1-Luc (*n* = 2), and a second group received a suspension of purified MS2RLP-Luc (*n* = 4). A noninjected animal was a negative control. Measurements were carried out at 5 hours up to 168 hours after systemic injection. (**b**) Quantitative measurement of luciferase. The top panel shows all the measurements from 5 to 168 hours, the bottom panel is an enlargement of the 5- to 24-hour period highlighting the differences between the two vectors exacerbated at 5 hours. Data are expressed in relative luminescence units. (**c**) *In vivo* analysis for YFP expression in ROSA 26 YFP mice after injection of purified suspensions of MS2RLP-Cre or rLV-EF1-Cre. YFP immuno-staining (green) on 20 µm thick cryosections of injected muscles of the left thigh. Nuclei were stained with Hoechst 33342 (blue). Mice were injected with either rLV-EF1a-Cre (A–D) or MS2RLP-Cre (E–G); internal negative control is shown (H). Phosphate-buffered saline–injected muscle were not stained (data not shown). Photographs are representative of stained sections obtained from each individual animal (see text). Enlarged areas (white rectangles) B, C, and D are from a (rLV-EF1a-Cre); and F, G, and H are from E (MS2RLP-Cre). Scale bars: A and E = 1 mm, B–D and F–H = 100 µm.

First, we checked caudal vein delivery using a purified preparation of MS2RLP (see methods). MS2RLP-Luc (1.7 × 10^11^ PP/ml) was employed to determine the kinetics of luciferase expression with systemic injection in mice. A suspension of purified integrative rLV-EF1-Luc vector (2.1 × 10^**8**^ TU/ml) was used as a positive control. Administration by the intravenous route induced a broad diffusion of the bioluminescent signal, which was quantified in the whole mouse body (except the tail signal). At 5 hours after intravenous injection of purified particles, we detected significant luciferase expression (818 relative luminescence units); the signal was still detectable 8 hours postinjection (234 relative luminescence units) and then disappeared at later times (Figure 4a,b, bottom panels, group MS2RLP-Luc). As expected for caudal vein injection, the bioluminescence signal was predominantly detected in the liver and spleen (Figure 4a). For comparison, the 5-hour time point measured in animals receiving the purified suspension of integrative rLV-EF1-Luc vector gave a barely detectable signal in the liver and spleen (Figure 4a). For rLV-EF1-Luc, the signal was significant at 72 hours and almost 7 days (168 hours) were needed to reach the maximum level observed with MS2RLP-Luc (Figure 4b). These kinetics parallel those obtained *in vitro* and further emphasized the explosive and brief expression obtained with MS2RLP mRNA delivery as compared to stable lentiviral expression.

To confirm the value of single-shot mRNA delivery, we examined the process with MS2RLP-Cre in ROSA26-YFP reporter mice.^32^ We locally injected MS2RLP-Cre (10^7^ PP in 5 µl) into the *biceps femoris* muscle of ROSA26-YFP reporter mice. Injected samples were collected from *biceps femoris* of five mice 7 days postinjection. As shown in Figure 4c, this model highlighted the efficiency of the nonintegrative MS2RLP-Cre system, which conferred expression of the YFP in a wide zone of injected muscle (Figure 4c, A, B, and C, as compared to nonaffected muscle zone H). MS2RLP-Cre spread in approximately 1 cm of the muscle, and this was comparable to the area reached with a purified integrative rLV-EF1-Cre vector used as a positive control (Figure 4c, E, F, and G). Altogether, *in vivo* data confirmed the potency of MS2RLPs to deliver functional mRNA locally or by systemically.

### MS2RLPs containing transcription factor mRNA promote a cell-fate shift in human MSCs

Cell therapy applications could benefit from the transient expression of differentiation factors. Thus, we assessed the ability of MS2RLPs to trigger a shift in cell fate by expressing two transcription factors involved in osteoblastic differentiation. *RUNX2* and *DLX5* are master transcription factors inducing skeletogenesis in mesenchymal stem cells.^33^ *DLX5* is a member of the distal-less HD protein family and has been implicated in the commitment of mesenchymal progenitors to the osteoblast lineage by the upregulation of *RUNX2* and *OSTERIX*. For *RUNX2*, we cloned the cDNA variant 1, type II isoform that is predominantly expressed in osteoblasts and is considered essential for osteoblastic differentiation.

In a first study, human osteosarcoma MG63 cells, exhibiting an immature phenotype reminiscent of MSCs,^34^ were used to assess the biological effect of *DLX5* or *RUNX2* mRNA transfer. MG63 were transduced with MS2RLP-*RUNX2* or MS2RLP-*DLX5* (50 × 10^3^ or 100 × 10^3^ MS2RLPs PP/cell) and cultured with or without the osteogenic differentiation factor BMP4.^35^ Retroviral vectors stably expressing the transcription factors were used as positive controls. In the presence of BMP4, and 10 hours after transduction, *DLX5* and *RUNX2* mRNA transfer induced the endogenous forms of *RUNX2* and *DLX5* respectively (Supplementary Figure S4a,b; dark blue). In cells transduced with MS2RLP-*RUNX2* or MS2RLP-*DLX5* and without BMP4, the *PTHR1* gene showed increased mRNA expression (Supplementary Figure S4c, dark blue), confirming that MS2RLP RNA delivery could induce transcription of osteoblastic genes.

**Figure 5:**
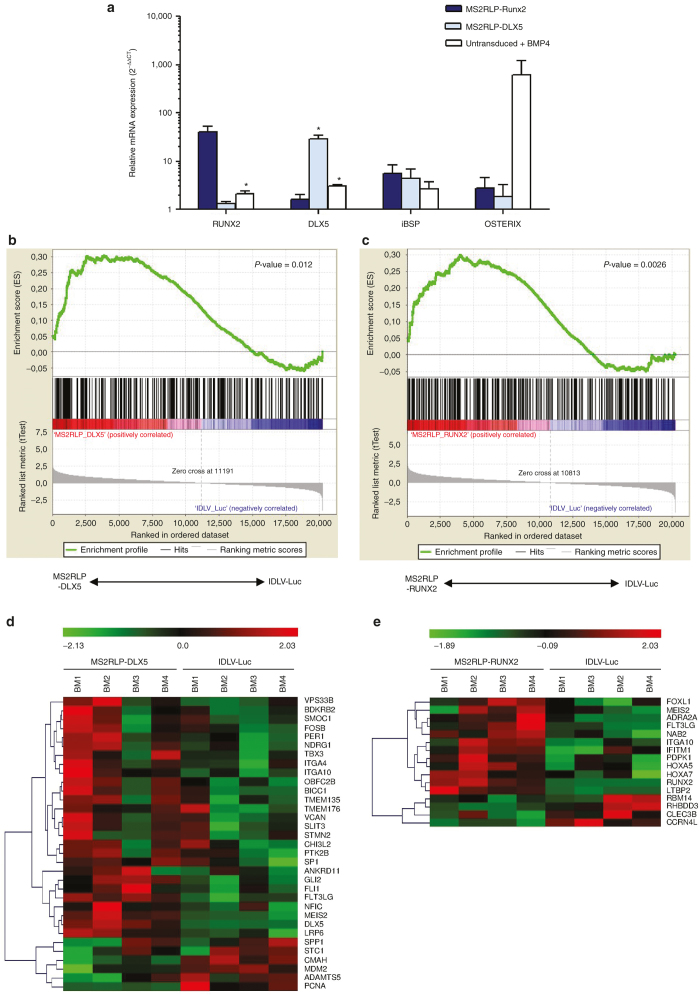
MS2RLP delivery of transcription factors promote a cell-fate shift in human MSC-I. (**a**) MS2RLP effect on osteoblastic differentiation of human MSC-I. Results for RT-qPCR analysis of the expression of gene osteoblastic in immature human mesenchymal stem cells (MSC-l) after MS2RLP-*RUNX2* or MS2RLP-*DLX5* mRNA transfer. MSC-l cells precultured in EGM2 medium were transduced with 50,000 PP/cell of MS2RLP-*RUNX2* (dark blue bars), MS2RLP-*DLX5* (light blue bars) or control cells with BMP4 (white bars). Following transduction, cells were maintained in αMEM supplemented with 2% FCS and mRNA expression of *RUNX2*, *DLX5*, *iBSP*, and *OSTERIX* was measured at 58 hours post-transduction. Significance was measured by one-sample *t*-test with the reference value fixed to 1, which corresponded to MSCs transduced with nonintegrative IDLV-Luc. Data are mean ± SD (*n* = 4) (*P < 0.05). (**b** and **c**) Gene Set Enrichment Analysis. Osteoblastic upregulated markers genes obtained after MS2RLP-*DLX5* mRNA transfer into MSC-l cells. Osteoblastic markers were enriched (*P* = 0.012) (**b**). Osteoblastic upregulated markers genes obtained after MS2RLP-*RUNX2* mRNA transfer into MSC-l cells. Osteoblastic markers were enriched (*P* = 0.0026) (**c**). (**d** and **e**) Representative heat maps highlighting osteoblastic genes with increased and reduced expression. The heat map shows the effect of MS2RLP-*DLX5* mRNA transfer in MSC-l with a reference of MSC-l transduced with lentiviral particles IDLV-Luc (**d**). The heat map shows the effect of MS2RLP-*RUNX2* mRNA transfer in MSC-l with a reference of MSC-l transduced with lentiviral particles IDLV-Luc (**e**). Red: genes with increased expression; and green: gene with decreased expression. Colour intensity represents the range of difference. MEM, Minimum Essential medium.

Next, we examined lineage commitment in highly immature primary human MSC-like cells (MSC-l)^36^ not expressing *RUNX2*.^37^ MSC-l cells were transduced twice at 30 hours intervals, with MS2RLP-*RUNX2* or MS2RLP-*DLX5* (50 × 10^3^ MS2RLP PP/cell, see methods). Controls were MSC-l transduced with IDLV-Luc or left untransduced and BMP4-treated, a condition known to consistently differentiate MSCs into functional osteoblasts.^38^ At 58 hours post-transduction, endogenous genomic *RUNX2* or *DLX5* mRNA level significantly increased (Figure 5a). In addition, the osteoblastic marker integrin-bone sialo-protein (iBSP) was upregulated in both conditions (Figure 5a). Conversely, the osteoprogenitor master factor *OSTERIX* showed no change in expression (Figure 5a), suggesting a commitment of MSC-l toward an early osteoblastic lineage stage.

To complete the study, we analyzed the MSC-l transcriptome following transduction and with IDLV-Luc as a reference. Principal component analysis showed that MS2RLPs expressing *RUNX2*- or *DLX5*-mRNA promoted the preferential expression of osteoblastic genes in MSC-l (Figure 5b,c). However, genes from MS2RLP-*DLX5-*modified cells were better clustered in the same branch than those from MS2RLP-*RUNX2*-modified cells (Figure 5d,e and Supplementary Figure S5), suggesting that MS2RLP-*DLX5* may be more potent in inducing changes in gene expression. Detailed analysis of significant gene expression changes confirmed the increase in *RUNX2*, *DLX5*, and *iBSP* levels (Supplementary Tables S2 and S3). We identified several osteoblastic markers among other upregulated genes, such as PTGS2 and FOSB in MS2RLP-*RUNX2* cells and BMP8, VDR, ENPP1, MEIS2 in MS2RLP-*DLX5* cells (Figure 5d,e and Supplementary Tables S2 and S3). Interestingly, we confirmed previous observation showing that *DLX5* induced *RUNX2* whereas *RUNX2* failed to induce *DLX5* (Supplementary Table S2; Supplementary Figure S5a).^39^ In addition, among upregulated genes by MS2RLP-*RUNX2*, we identified *STMN2,* a gene induced in adipose and bone marrow-derived stromal cells under pro-osteoblastic differentiation conditions.^40^ We confirmed the consistent and early upregulation of *STMN2* mRNA expression after BMP4 addition on highly immature mesenchymal stromal-like cells (Supplementary Figure S5b,c). Finally, we identified a series of common genes with significant expression changes upon MS2RLP-*RUNX2* or -*DLX5* transfer and BMP4 addition, conditions known to consistently differentiate MSC-I into functional osteoblasts^38^ (Supplementary Figure S6a). Regarding shared upregulated genes in BMP4-induced cells and under the MS2RLP-*RUNX2* or -*DLX5* condition, the bone/skeletal development process was clearly highlighted by Gene Ontology terms (Supplementary Figure S6b), especially for MS2RLP-*DLX5* and BMP4 conditions (Supplementary Figure S6b). Overall, MS2RLP-*RUNX2* or MS2RLP-*DLX5* consistently committed human primary MSC-I toward an early osteoblastic lineage, confirming that MS2RLPs could contribute to promoting cellular reprogramming (Supplementary Tables S4 and S5).

## Discussion

In the present report, we described a versatile and efficient cell-transfer system for mRNA or other types of RNAs. Our results showed that exploiting MS2-Coat and its cognate 19-nt stem-loop, led to significant RNA transfer via modified lentiviral particles in all types of cells *in vitro* or *in vivo* (Figures 2–5). As compared to other retroviral platforms, transfer efficiency was high, without any requirement for a selection process^10,41^ (Figures 2–4). In a therapeutic perspective, an advantage of the MS2RLP system is its ability to utilize lentiviral platforms already validated in clinical settings. Moreover, the RNAs transferred by the MS2RLPs are directly expressed into the cytoplasm, which reduces the risk of integration, an important safety consideration for human use (Figure 2e). This characteristic is in contrast to transient episomal DNA vectors, such as IDLV, which lead to some integration at low frequency.^42^ Although our studies have been performed with lentiviral vectors other retroviruses could be similarly modified. Introducing MS2-Coat sequence within the p12 protein from a gammaretrovirus-derived vector^6^ also permitted transfer of RNAs harboring the 19-nt stem-loop (Supplementary Figure S7). In addition, like for other vectors, it could be possible to adapt MS2RLPs to a cell type by selecting the envelope exposed at their surface.

The high efficiency of RNA transfer by the MS2RLPs is most likely related to an increased ratio of RNA to particle content, resulting in directly detectable and significant biological activity (Figure 2). This is extremely valuable when seeking editing or reprogramming functions, especially if a combination of gene products is required.^43^ Additionally, RNAs from MS2RLPs are rapidly bioavailable (Figures 2b and 4b), leading to high and short-term expression of the transferred messenger. This characteristic is of great value for genome engineering. In lentiviral transgenesis, the early expression of the transgene following egg-injection is expected to reduce the risk of genome chimerism in the progeny.^44^ Short-term and efficient RNA expression would also be valuable in several other applications, such as vaccination and immunotherapy.^45^ However, as highlighted by the comparison of the kinetics of expression of luciferase and GFP (Figure 2a,b compared to c and S2), the half-life of the transferred protein could be a concern as was shown recently with fluorescent proteins.^30^ Therefore, the biochemistry of the transferred protein is expected to affect the requirements for designing a useful vector. Several methods could be used to modulate the level of protein expression, including the stabilization of the RNA, with modification of nucleotide usage,^45^ or by using defined 5′ and 3′ UTRs.^11^ For a more sustained expression, viral subgenomic replicons would offer a relevant alternative. Accordingly, we showed MS2RLP-based transfer of an HCV replicon in Huh7.5 cells (Figure 3). To build new transfer-platforms, several alternative RNA-replicons exist. Recently, replicons derived from Venezuelan equine encephalitis virus (VEE) and expressing reprogramming genes were shown to support iPS formation.^43^ To ensure safety after obtaining the intended biological effect, eradication of the reprogramming replicon is expected and could be triggered by use of cell-fate specific miRNA.^46^ Nevertheless, a case-by-case analysis could be useful to evaluate the innate immune response of the target cells, since it could block replication of the replicon.^47^ For example, we were unsuccessful in expressing reporter genes from an HCV-replicon transferred by MS2RLP in primary human MSC (data not shown).

MS2RLPs are versatile vectors as demonstrated here by *in vitro* transfer of mRNAs in cell lines, human iPS, primary human mesenchymal cells and mouse liver or muscles *in vivo*. While we did not compare MS2RLP to all RNA-transfer methods, our results showed that human primary CD34^+^ cells, a clinically relevant target not easily transfected using electroporation or chemical transfection, could express high level of a transgene following MS2RLP transduction (Figure 2c and Supplementary Figure S2b). As for other cell types, CD34^+^ cells expressed the transgene at a level similar to that reached by IDLV (Figure 2c1–c2). Yet, the kinetic was slightly different, MS2RLP giving a higher and faster transgene expression at early time points. Notably, the expression induced by MS2RLP in CD34^+^ cells was not conditioned by the addition of cytokines.^29^

The diversity of RNAs that could be transferred by MS2RLPs is only dictated by the presence of the 19nt MS2 stem-loop. Hence, the molecular structure of transferred RNA is adaptable and amenable to regulation with some tools already available.^7^ Here, we have shown that mRNA and RNA-replicon were mobilized (Figures 1–4), a property which could no doubt be extended to LncRNA or circRNA as modulators or regulators of gene expression.^48,49^ The transfer of interfering RNAs will need more investigations because the pathway of miRNA maturation involves both nuclear and cytoplasmic steps as well as cleavages, which can separate the recruiting sequence from the active part of the molecule. An aspect we have indirectly explored is the size capacity and limits of the transferred RNA. Our experiments showed that the short GFP sequence and HCV replicon, of approximately 10 kb (Figures 2 and 3), could be transferred efficiently. This covers most of the needs in the field of RNA transfer. Similarly, by recruiting the RNA independently of dimerization (Figure 1) and with more than four molecules per particle (Supplementary Figure S1b), MS2RLPs should permit the cotransfer of different species of RNA. For the reprogramming experiments, several different osteogenic factors could be used, and the opportunity to sequentially deliver these factors through MS2RLPs might be very valuable. Among potential applications are gene-editing methods.^3^ The opportunity to get highly efficient Cre recombinase activity after mRNA MS2RLP delivery (Figures 2d and 4c) paves the route to transfer of other systems such as those based on CRISPR/Cas (a preliminary experiments is given in Supplementary Figure S2f).^3^ For the transfer of the CRISPR/Cas9, the multiplexing of several species of RNAs, one coding Cas9 and others the sgRNA, would be needed. Moreover, it will be a complex challenge to obtain optimal stoichiometry following encapsidation of the different RNA species into the same particles.

Considering *in vivo* applications, our results further illustrate the efficacy of MS2RLPs for the delivery of genetic information. For vaccination, besides the expression of immunogenic proteins by RNAs, immune response could be improved by transferring subgenomic replicons. Expressing viral nonstructural genes could contribute to the clearance of cells infected by the native virus. By mobilizing an HCV replicon from Huh7.5 cells (Figure 3), we showed that MS2RLPs were also compliant in terms of production and could be eligible for *in vivo* local production of vaccinating particles.^50^ In the mouse muscle *in vivo,* Cre expressing MS2RLPs allowed for a spread of the excision over 1 cm (Figure 4c). Such diffusion is broad and equivalent to that obtained with direct injection of nonenveloped vectors such as adeno-associated virus-based vectors.^51,52^ Systemic injection, while it mainly directs the vector load in the liver and spleen (Figure 4), offers a relevant alternative to delivering active RNAs. As mentioned above, organ specificity could be addressed by choosing the most adapted cell-penetrating envelope.

We also applied the system in a cell therapy approach, primary MSC-l (Figure 5 and Supplementary Files). Our data confirmed that transfer of bone specific transcription factor via MS2RLPs promoted osteogenesis induction (Figure 5). However, the protocol evaluated here proved to be limited to the priming of the MSC-l. We nevertheless identified *OSTERIX*, *STMN2* and other genes as possible targets to trigger a more profound fate-shift toward the osteoblast phenotype. There is currently no definitive recipe to achieve this process in culture.^53^ Adapting MS2RLPs to high-throughput screening procedures will help to identify key factors and protocols for controlled administration of selected genes.

In conclusion, thanks to high titers on primary cells as well as *in vivo*, the new MS2RLPs described here, offer a significant and important alternative in the field of RNA delivery. Because of their versatile nature in packaging various types of RNAs, MS2RLPs have broad potential for application, including stem cell-differentiation, immunotherapy and genome editing.

## Materials and Methods

### Packaging vector and plasmid expression constructs

#### HIV-I-derived MS2RLP.

An MS2 containing HIV-I-derived vector was generated by substituting the NC’s second zinc finger (ZF2) domain with the MS2 coat protein. The ZF2 domain was removed from p8.74 lentiviral plasmid using assembly PCR. The upstream region of the deletion site was amplified by using P8.74bZF-for: CCgCggCCgCgTTgACgCgCA (containing a Not-I site) and P8.74bZF-rev: CAgTgTTAACgCCCTTTTTCCTAgg (containing a HpaI restriction site). The downstream region of the deletion site was amplified using P8.74aZF-for: ATgCggCCgCgTTAACACTgAgAgACAgg (with a Not-I site) and P8.74aZF-rev: ggCTgATATCTAATCCCTggTgTCTC (containing a EcoRV site). After subcloning into PGEMT-easy plasmid, vectors were NotI/EcorV digested and reassembled into NotI/EcoRV digested p8.74 plasmid to generate p8.74∆ZF vector (ΔZF). The MS2 Coat protein coding sequence was amplified from LMNI-MS2-NLS-GFP plasmid, kind gift of P.D Bieniasz (Aaron Diamond AIDS Res. Center, New York), using HpaI containing primers MS2Coat-HpaI-for: TTgTTAACATggCTTCTAACTTT, and MS2Coat-HpaI-rev: CCgTTAACgTAgATgCCggAgTT and was inserted into p8.74∆ZF digested by HpaI leading to an in-frame cloning and generating p8.74 ∆ZF_MS2Coat (ΔZF_MS2coat) Coat vector (Figure 1).

#### Engineering mRNA.

To enable the mobilization of mRNA into lentiviral particles, 12 repeats of the MS2 stem-loops were inserted into the expression vector pcDNA3.1 (Life Technologies SAS, Saint Aubin, France) containing the luciferase gene reporter. PCR and specific primers were used to obtain the entire luciferase Firefly sequence by PCR (Table 1), which was cloned into pcDNA3.1(-) vector (Life Technologies SAS) at the HindIII and KpnI sites to generate pcDNA-luciferase (pLuc). To generate pcDNA-luciferase-MS2-12X (Luc_MS2_12X), fragment containing 12 repeats of MS2 was obtained by digestion of pGemTeasy-MS2-12X plasmid with BamHI. The obtained fragment was blunted with DNA polymerase I large (Klenow) fragment (5,000 units/ml; New England Biolabs, Evry, France) and inserted into 3’ of luciferase gene in pcDNA-Luc plasmids previously digested with KpnI and treated with DNA polymerase I large (Klenow) fragment (Figure 1).

In parallel, a pcDNA-EF1-Cre-MS2_12X plasmid was generated. To construct this vector, a pCMV-luciferase sequence was deleted from the pcDNA-luciferase-Vimentin-MS2_12X vector and substituted by a pEF1-Cre sequence amplified from a previous construct. The plasmid was generated by using the In-Fusion HD cloning kit (Clontech Laboratories). To avoid recombination, MS2 stem-loops-containing plasmids were grown at 32 °C.

For integrative and integrative-deficient lentiviral vectors, we used three different self-inactivating expression vectors, pLV-EF1-GFP, pLV-EF1-Luc, and pLV-EF1-CRE, based on the backbone described by Dull *et al*.^26^ These vectors encode the GFP, the luciferase or the CRE recombinase, respectively under control of the human elongation factor 12α promoter (EF1a).

#### MS2 tagging of HCV RNA.

MS2 stem-loops from pSL-MS2-24 plasmid (gift from R.H. Singer, Albert Einstein College of Medecine, Bronx, New York under MTA) were introduced into the JFH-1 HCV subgenomic replicon coding sequence. A fragment containing 24 repeats of the MS2 recognition RNA motif was obtained by EcorRV/BamHI digestion and sub-cloned into pGEMT-easy vector. Smaller fragments containing 6 and 12 repeats of MS2 motif were amplified by using MS2-BamHI-for: ACCCgggCCCTATATATggATCC forward primer and the reverse primers MS2-BamHI-6X-rev: ATggATCCgTgATTCCCCg or MS2-BamHI-12X-rev: TAggCAATTAggATCCTTAggAT containing BamHI restriction site. Resulting PCR products were sub-cloned into pGEMT-easy vector. MS2 repeats harbouring pGEMT constructs were then BamHI-digested, Klenow treated, and inserted into pSGR-JFH1-Neo subgenomic replicon coding plasmid at the PmeI restriction site (Figure 3). As above, MS2 stem-loops-containing plasmids were grown at 32 °C.

### Viral vector production and quantitation

#### Amphotropic pseudotyped lentiviral particles.

Productions were performed in 293T cells (ATCC CRL-11268) seeded at 4.2 × 10^6^ cells per plate into a 10-cm dish and grown overnight in Dulbecco’s modified Eagle medium (DMEM) Glutamax (Life Technologies, Carlsbad, CA) supplemented with 10% heat-inactivated fetal calf serum, 100 µg/ml Stretomycin, 100 U/ml Penicillin and placed at 37 °C in a humidified atmosphere of 5% CO_2_ in air. Fugene 6 transfection reagent was used to transfect cells with 1 µg p8.74ΔZF-MS2-coat, 1 µg of pCMV-Env encoding 4070A amphotropic envelope, 80 ng pEGFPC1 for normalization and 2.52 × 10^−13^ mole of Luc_MS2_12X. After 8 hours, the medium was removed and cells were washed with phosphate-buffered saline (PBS), then fresh medium was added. After 48 hours, particles-containing supernatants were harvested and filtered through a 0.45-µm pore filter (Millipore, Saint Quentin en Yvelines, France).

#### VSV-G-pseudotyped lentiviral particles.

Productions were performed in a 10-layer CellSTACK (6,360 cm^2^, Corning) using 293T cells. The tri-transfection mix was composed of three plasmids: pENV carrying the VSVG envelope, p8.74 (WT) or p8.74 ΔZF_MS2coat or p8.74 mutated in the D64L to produce (IDLV), and pLV-EF1-cDNA/pMS2-cDNA. Twenty-four hours post-transfection by standard calcium phosphate procedure, the supernatant was discarded and replaced by fresh nonsupplemented DMEM. Cells were incubated at 37 °C in a humidified atmosphere of 5% CO_2_ in air. After medium change, the supernatant was collected. Fresh nonsupplemented DMEM was added and the cells were incubated prior to further harvest. Each harvest was clarified by centrifugation for 5 minutes at 3,000*g* before being microfiltered through a 0.45-µm pore-size sterile filter unit (Stericup, Millipore). The whole set of harvest were then pooled to supply the crude harvest.

#### Viral vectors concentration and purification.

The crude harvest was first concentrated by tangential flow ultrafiltration with polysulfone hollow-fiber cartridges. The supernatant was then diafiltered against DMEM buffer, and retentate was recovered and further concentrated on ultrafiltration disposable units.

#### Functional particle quantification by qPCR.

Transduction unit titration assays were performed as follows. HCT116 cells were seeded in 96-well plate in 100 µl of DMEM supplemented with 10% heat-inactivated foetal calf serum, 100 µg/ml Stretomycin, 100 U/ml Penicillin. Twenty-four hours later, five serial dilutions were performed with each vector sample and an rLV-EF1-GFP internal standard. The cells were transduced in presence of 8 µg/ml Polybrene (Sigma). For each sample series, one well of nontransduced cells is added as control. Three days post-transduction, cells were trypsinized and the titer (transducing units/ml) was determined by qPCR after extraction of genomic DNA using the Nucleospin tissue gDNA extraction kit (Macherey-Nagel, Hoerdt, France). The titer determined in transducing units per ml (TU/ml) using qPCR was normalized by an internal standard whose titer was previously determined by FACS.

#### Physical particle quantitation by p24 ELISA.

The p24 core antigen is detected directly on the viral supernatant with use of a HIV-1 p24 ELISA kit (Perkin Elmer, Courtaboeuf, France). The reaction is based on an antigen capture recognized with a biotinylated polyclonal antibody against HIV-1 CAp24. The resulting complex is revealed by a streptavidin–horseradish peroxidase conjugate after incubation with ortho-phenylenediamine-HCl, producing a yellow colour proportional to the amount of CAp24 captured. The absorbance of each well is measured; quantification is obtained as compared to a standard curve established with reference HIV-1 CAp24 antigen samples, on a microplate reader. The viral titer expressed in physical particles per ml is calculated from the amount of p24 knowing that 1pg of p24 corresponds to approximately 10^4^ physical particles.

#### Quantitative real-time RT-qPCR.

To assess mRNA mobilization into lentiviral particles, RNA was extracted from 140 µl lentiviral particles containing supernatants by use of the QIAamp viral RNA Mini-kit (Qiagen, Courtaboeuf, France). In total, 4 µl RNA treated with TURBO DNAse (2 U/µl) was used for cDNA synthesis with the Superscript First-strand Synthesis System (Life Technologies). Luciferase RNA copies were quantified by RT-qPCR, with a Light Cycler480-II system (Roche, Boulogne-Billancourt, France). RT-qPCR amplification: 2 µl of reverse transcriptase product, 1× SYBR Green I master mix (Roche), 10 µmol/l of each primer and RNase-free water to a final volume of 20 µl was quantitated by 5-minute preamplification at 95 °C, 45 cycles: 10” at 60 °C and 10” at 72 °C, followed by melting curve and cooling steps. PCR amplification was obtained with primers allowing specific amplification of the luciferase, forward, 5′-CAACTgCATAAggCTATgAAgAgA-3′ and reverse, 5′-ATTTGTATTCAgCCCATATCgTTT-3′. The RNA copy number was calculated by an external standard curve of serial dilution of a pBullet-Luc plasmid.

#### Quantitative real-time RT-qPCR on serial dilution.

Since the CAp24 normalization could be someway misleading, we designed an unbiased experiment to estimate MS2RLP RNA-content. Supernatant from cells producing the MS2RLP or a standard lentiviral vector (ILV) were serially diluted, six serial one-half dilutions from crude supernatant, and the amount of RNA per sample was measured by RT-qPCR (see above for RT-qPCR). From the data, we draw a curve and derived their respective equation (Supplementary Figure S1b). The curves are theoretically expected to converge to the intercept and their respective slopes are proportional to the amount of RNA per particle. The ratio calculated using MS2RLP slope over ILV slope gives the factor of difference in RNA content between the two types of particles, independently of the structure of the particles. The experiments were repeated independently three times in triplicate.

### Electron microscopy

Transmission electron microscopy was performed at IMPMC (UMR 7590, CNRS-UPMC-IRD-MNHN, Paris, France). For negative staining, samples produced at about 10^12^ PP/ml were diluted 50-fold and 5 μl were dropped onto plain carbon grids, blotted, and immediately stained for 1 minute with 5 μl of 2% uranyl acetate before blotting again. The sample was allowed to dry before observation under a LaB6 JEOL JEM2100 electron microscope (×52,800 magnification). Data were digitally recorded by use of a 2k × 2k Gatan camera.

### Luciferase assay and luciferase kinetics

On the day before transduction, 60,000 293T cells were seeded in 96-well plate. The day of transduction, test samples of MS2RLP were applied to cells with or without 3 µg/ml puromycin. This translation inhibitor was added 1 hour before transduction. For 4070A envelopes transduction was realized in presence of Polybrene (5 µg/ml, Sigma-Aldrich, Saint Quentin Fallavier, France) and under centrifugation for 1 hour at 1,900 rpm and 37 °C. Following transduction, luciferase expression was determined by use of the Bright-Glo luciferase assay (Promega, Charbonnières-les-Bains, France) and a Centro LB 960 luminometer (Berthold Technologies). The assay was performed in triplicate.

For the kinetics, HCT116 cells were seeded in six-well plate and incubated 24 hours at 37 °C in 5% CO_2_. Transduction by ILV, IDLV, or MS2RLP was carried out in presence of 4 µg/ml Polybrene with 2 × 10^5^ physical particles/cell. The transduction-supernatant is removed 5 hours later. From 4–24 hours post-transduction, cells were harvested and luciferase expression was analyzed using the One-Glo Luciferase assay (Promega) as above. The assay was performed in triplicate.

### Cre recombinase *in vitro*

HCT116 cells were transduced by an ILV-EF1-Lox-dsRed-Lox lentiviral vector at a multiplicity of infection of 20 with 4 µg/ml Polybrene. Six days later, polyclonal cells were cloned by limit dilution by seeding 0.5 cells/well in 96-well plate. Clone #14 was selected by FACS (MACS Quant VYB, Miltenyi, Paris, France). Polyclonal or clone #14 HCT116-Lox-dsRed-Lox cells were transduced with IDLV-CRE or MS2RLP-CRE with 2 × 10^5^ PP/cell. 14 days post-transduction, the dsRed expression was analyzed by FACS. The assay was performed in triplicate.

### Hematopoietic cell transfection and transduction

Hematopoietic cord blood cells were obtained from the “Fondation Générale de Santé” and the “Assistance Publique-Hôpitaux de Paris”. CD34^+^ cells were purified using CD34 microbeads (Miltenyi Biotec) and the autoMACS Pro separator (Miltenyi Biotec). For transduction and transfection experiments, cells were grown in X-VIVO20 (Lonza, Levallois, France) complemented with cytokines (Peprotech), including hSCF (100 ng/ml), hTPO (100 ng/ml), hFLT3L (100 ng/ml), and hIL3 (60 ng/ml).

The nonintegrative GFP vector (IDLV-GFP) supernatant was produced by transient transfection of HEK293T cells in 10-cm dishes, using Fugene 6 (Promega). Plasmids were a self-inactivating (SIN) and Tat-independent lentiviral vector encoding GFP under the control of the EF1α promoter/enhancer (LTGCEGFP1), and three packaging plasmids including HPV275D64A (HIV1 gag/pol with a D64A mutation in integrase), p633 (expressing rev), and ΨN15 (VSVG).^54^ IDLV supernatants were collected 48 hours after transfection, filtered through a 0.45-µm filter, concentrated ≈1,000-fold by ultracentrifugation and stored at −80 °C; ELISA CAp24 concentration was 13.4 ± 0.4 µg/ml.

The GFP RNA vector (MS2RLP-GFP) was produced by transient transfection of HEK293T cells in 10-cm dishes, using Fugene 6. Plasmids transfected consisted of the GFP encoding plasmid pVAX2-EGFP-MS2-12X, and three packaging plasmids including p8.74_ΔZFMS2Coat, p633 (rev) and ΨN15 (VSVG). pVAX2-EGFP-MS2-12X was made by subcloning EGFP-MS2-12X from pBullet-EGFP-MS2-12X in pVAX2. It encodes EGFP under the control of the Human cytomegalovirus immediate-early (CMV) promoter and has a bovine growth hormone polyadenylation signal. pVAX2 was obtained by replacing the cytomegalovirus promoter of pVAX1 (Life Technologies) by that of pCMVβ (Takara), as described.^55^ VLP supernatants were collected 48 hours after transfection, filtered through a 0.45-µm filter, concentrated ≈1,000-fold by ultracentrifugation and stored at −80 °C; ELISA CAp24 concentration was 27.4 ± 3.2 µg/ml.

The GFP mRNA was obtained from Trilink Biotechnologies. It carries an antireverse cap analog at the 5’ end, it is polyadenylated, and is modified with 5-methylcytidine and pseudouridine to decrease nuclease activity and Toll-like receptor activation, mimicking a fully processed mature mRNA. Transfection used the 4D-Nucleofector X Unit and the P3 Primary Cell 4D-Nucleofector X Kit (Lonza) or the TransIT-mRNA Transfection kit (Mirus Bio). A control plasmid expressing GFP (pmaxGFP) is included in the Nucleofector X kit.

GFP expression was determined by flow cytometry using the MACSQuant Analyser (Miltenyi Biotec) and the FlowJo software (Tree Star) for vector transduction or the FACSCanto II and the Diva software (Becton Dickinson) for plasmid and mRNA transfection.

### GFP expression in Hela cells

50 × 10^3^ Hela cells were seeded in six-well plates 24 hours before transduction. Approximately 1 × 10^8^ physical particles per well were used. After 24 hours, cells were washed with saline solution and analyzed by flow cytometry (FACS) or cultured for a total of additional 7 days to follow GFP expression.

### Transduction of human induced pluripotent stem cells

The BBHX8 hiPSC line was maintained on Matrigel-coated culture wells (BD Biosciences, San Jose, CA) in mTeSR1 (Stemcell Technologies, Vancouver, BC, Canada) at 37 °C in 5% CO_2_ incubator with daily medium change and by using Gentle Cell Dissociation Reagent (Stemcell Technologies) for cell passaging. Three days before transduction, 200 × 10^3^ cells were seeded in 12-well plate. Transduction was performed for 24 hours with different amount of viral particles, in ng of CAp24, and with cells at 60–70% confluence. Before FACS analysis, cells were detached using TrypLE (Life Technologies).

### HCV RNA *in vitro* transcription

After XbaI linearization of plasmids, modified and control HCV replicons RNAs were synthetized *in vitro* by transcription using RiboMAX large-scale RNA production system T7 (Promega). After DNAse treatment, RNAs were purified with RNA clean-up kit (Macherey-Nagel) before transfection.

### HCV RNA transfection and quantitative real-time PCR

*In vitro* synthetized HCV replicons, RNAs were transfected into Huh7.5 cells by electroporation: trypsinized cells were washed in PBS, and 2 × 10^6^ cells were resuspended in 1 ml PBS and placed in 0.4-cm electroporation cuvettes (Biorad). Electroporation with a 950 μF and 260 V pulse in a Genepulser system (Biorad). After electroporation, cell suspension was transferred in 10-cm plates containing medium and incubated at 37 °C under standard conditions. Two days after transfection, cells were selected with 1 mg/ml geneticin.

Total RNAs from transfected Huh7.5 and Huh7.5 HCV were extracted (Nucleospin RNAII kit, Macherey–Nagel), and 0.5 µg of RNA was used for cDNAs synthesis (Super-Script First-Strand II Synthesis System, Invitrogen). JFH1 RNA quantification was performed by qPCR with JFH1 and β-actin-specific primers (Supplementary Table S1) using SYBRGreen incorporation (SYBRGreen I MASTER). Cycling conditions were 5 minutes preamplification at 95 °C, 45 cycles (10” 95 °C, 10” 60 °C, 10” 72 °C) (Light-Cycler-480-II, Roche).

### MS2RLP-HCV production and colony formation assay

For HCV replicon RNA mobilization, 2.5 × 10^6^ Huh7.5 HCV cells harboring JFH1-derived subgenomic replicons were transfected using FuGENE-6 (Promega) with 1.5 µg Gag-Pol coding plasmids, 1 µg of pCMV-Env Ampho plasmid. Supernatant from transfected cells were harvested and filtered (0.45-µm filters, Millipore) at 24, 36, 48, and 60 hours post-transfection. Naive Huh7.5 was transduced using 2 ml of filtered supernatants. Transduction was facilitated by 45 minutes spinoculation at 1,200 × *g* with 5 µg/ml polybrene (Sigma-Aldrich). Transduced cells were selected in 1 mg/ml geneticin 48 hours after transduction. Resistant clones were fixed in 2% paraformaldehyde, crystal violet stained and counted, using ImageJ cell counter pluggin. Results were obtained from four independent experiments performed in duplicates. After transduction, some clones were expanded forfurther analysis, by HCV RNA-specific RTqPCR or western blotting.

### Bioluminescence kinetics in mice

Seven-week-old Balb/c male mice (Janvier Labs, Saint Berthevin, France) received 200 µl of various vector suspensions by injection in the tail vein with 29G syringes. Injections were performed under a PSM II, according to a protocol validated by the local ethic committee. Animals were housed in ventilated racks in IVC cages in accordance with current European regulations. Two mice were given a suspension of purified Integrative lentiviral vector rLV-EF1-Luc, and a group of four animals received a suspension of purified particles MS2RLP-Luc and another group of four animals received a standard suspension of particles NILP-MS2-Luc. A noninjected animal served as negative control for background. Mice were anesthetized by continuous inhalation of 1.5% Isoflurane in O_2_-enriched air. Each measurement of luciferase was carried out 15 minutes after intraperitoneal injection of 300 mg/kg D-luciferin (Promega) with an Andor camera, image acquisition with the Solis software. The acquisition time was 5 minutes, and resulting images were processed and standardized with ImageJ software (U.S. National Institutes of Health, Bethesda, MD, http://imageJ.nih.gov/ij/ , 1997-2015).

### *In vivo* YFP expression in ROSA 26 YFP reporter mice after injection of MS2RLP-Cre or rLV-EF1-Cre

Eleven ROSA 26 YFP reporter mice^32^ were injected with ILV EF1a CRE (*n* = 5), MS2RLP (ARN) CRE (*n* = 5), or PBS (*n* = 1). Mice were anesthetized by continuous inhalation of 1.5% Isoflurane in O_2_-enriched air. A 5-mm incision of the skin above the left thigh was performed to visualize the *biceps femoris*. Five microliters of concentrated vectors or PBS were manually injected in a single injection site in the *bicept femoris*. The total dose injected corresponded to 3 µg of CAp24 and 0.5 µg for MS2RLP (ARN) CRE and ILV EF1a CRE vectors respectively. At 7 days after injection, the complete left thigh of mice were removed and fixed overnight in 4% paraformaldehyde in 1× PBS. Fixed tissues were cryoprotected in 15% sucrose in 1× PBS for 48 hours, frozen at −50 °C in isopentane and sliced in 20-µm-thick sections. The section axis was parallel to the global orientation of muscle fibers in the biceps femoris. YFP expression was observed after immunostaining as described previously^56^ with rabbit anti-GFP primary antibody (1:1,000; Molecular probes) and Alexa-fluor 488 goat anti-rabbit IgG as secondary antibody (1:1,000; Invitrogen). Nuclei were stained with Hoechst 33342. Photographs of whole sections were acquired with a Hamamatsu NanoZoomer scanner (20× resolution) and enlargement of selected areas were photographed with a Leica DM5000 epifluorescent microscope equipped with a Leica color camera.

### mRNA transfer study in primary bone marrow cells mesenchymal stem cells

#### MSCs transduction with lentiviral particles.

The study involved human bone marrow cells isolated from femoral heads from four healthy volunteers undergoing orthopaedic surgery. The study followed the ethical guidelines of the University Hospital of Tours (Tours, France) and was approved by the ethics committee Comité de protection des personnes (Tours - Région Centre (Ouest-1)). Cells were cultured in endothelial growth medium 2 (Promocell, Heidelberg, Germany) for 14 days. Three days before the transduction, cells were seeded in 12-well plates at 5,000 cells/cm^2^ and maintained in EGM2 medium. Transduction involved 50 × 10^3^ viral particles per cell (MS2RLP-*RUNX2*, MS2RLP-*DLX5*, or IDLV-Luc produced by Vectalys) with Polybrene (5 µg/ml), and cells were cultured in αMEM (Minimum Essential medium) supplemented with 2% fetal calf serum. After 12 hours, medium was removed and replaced. Thirty hours after the first transduction, cells were transduced again as above. At 10 and 58 hours, cells were washed three times with PBS and harvested.

#### RNA extraction.

Total RNA was extracted with RNeasy microkit (Qiagen) including a DNAse treatment. Extracted RNA was eluted in 15 µl RNase-free water. RNA concentration and quality was determined with Experion Automated Electrophoresis System (BioRad, Marnes-la-Coquette, France) and Nanodrop spectrophotometer (Thermo Scientific).

#### Real time qPCR.

First-strand cDNA was prepared using the High capacity reverse transcription kit (Applied Biosystems, Saint Aubin, France) with 100 ng total RNA and random primers. The expression of osteoblastic transcription factor *RUNX2*, *DLX5*, *iBSP*, and *OSTERIX* was evaluated by real-time quantitative PCR with EvaGreen incorporation (SsoFast EvaGreen kit, CFX96 Real-time PCR detection system, Biorad). PCR reaction: 4 µl of reverse transcriptase diluted at 2:1 in RNAse-DNAse-free water, 1× SsoFast EvaGreen kit, 10 µmol/l of each primer and RNase-free water to a final volume of 10 µl. Three minutes preamplification at 95 °C, 40 cycles of 10” at 95 °C and 30” at 60 °C. The *PPIA* gene was used as a normalization control.

#### cDNA microarray analysis.

Double-stranded cDNAs were prepared from 25 ng of total RNA with ovation PicoSL WTA system v2 kit (Nugen Leek, The Netherlands). The fragmented end-labeled cDNA was hybridized to GeneChip Human Gene 1.1ST array strip (Affymetrix, High Wycombe, UK) for 20 hours at 48 °C. After hybridization, the chips were stained and washed in a GeneChip Fluidics Station 450 (Affymetrix) and scanned using a GeneChip Array scanner (Affymetrix). The raw file generated contained expression intensity data and was used for analysis.

#### High-throughput studies.

Principal component analysis was performed for global unsupervised analysis. Supervised analyses for generation of each specific gene expression signature were obtained by using of Partek Genomics Suite software with the sample-paired *t*-test that matched each donor treatment. Gene expression signature was carried out for fold changes in expression > |1.2|, *P* < 0.05.

Differentially expressed genes were hierarchically clustered using the TM4 Microarray Software Suite^57^ with the Euclidian distance metric and average linkage method. Clustering and heatmaps involved TM4 Software Suite. Gene expression data with log values < 3 were not considered. Differential expression analysis involved a linear model with an empirical Bayes method to moderate the standard errors of the estimated log ratio changes. ConsensusPathDB-human (http://cpdb.molgen.mpg.de) served for functional annotation analysis with gene ontology terms.^58^ Venn diagrams were obtained with http://bioinformatics.psb.ugent.be. The Gene Set Enrichment Analysis^59^ (GSEA Broad Institute, Cambridge, MA) software was used for analyzing the median *t*-test of MS2RLP-*RUNX2* or MS2RLP-*DLX5* to the IDLV-Luc control, with a home-made gene list of osteoblastic markers.

### Statistical analysis

Data from three or four independent experiments in triplicate were analyzed. Data are expressed as mean−SD; Wilcoxon-Mann-Whitney was used to analyze the data.

## Notes

Supplementary Figures S1: A) Analysis of GAG precursors maturation. 25 ng P24 from MS2RLP or ILV suspension were directed loaded onto the SDS-PAGE. The blot was probed with an anti-P24 antibody. B) Curves for the measurement of RNA copy number per MS2RLP particle. For this we collected supernatant from cells producing the MS2RLP or a standard lentiviral vector (ILV). The supernatant was then serially diluted, 6 serial one-half dilutions, and the amount of RNA per sample was measured by RT-qPCR. For this estimation, we used the slope calculated from the experimental raw data, fixing that the curve should pass through the intercept. A theoretical curve would have had an equation such as: y = 2x for ILV and y = 2nx for MS2RLP, where x is the increment of particle number, where 2 is the number of RNA molecule in one particle and where n is the corrective factor reflecting the RNA content MS2RLP. The ratio between the two slopes is R = (145.79/52.041) = 2.8.

Supplementary Figures S2: A) Kinetics of luciferase activity upon MS2RLP transfer into HCT116 cells. HCT116 cells were transduced with MS2RLP-Luc (20 pg of Cap24/cell). At various times post-transduction (4H to 48H), cells were extensively washed, lysed, and luciferase activity was measured. B) GFP expression into hematopoietic cells transfected with GFPmRNA. 300,000 CD34+ cells were transfected with 0.25, 0.5, 1.0 or 1.5 µg of the GFP mRNA using the TranIT-mRNA transfection kit one day after cell thawing. GFP cell expression was analyzed 12, 24, 36 and 48 hours later by flow cytometry. C) GFP expression into Hela cells transduced with MS2RLPs. Hela cells were transduced with 108 MS2RLP-GFP. 24 hours, 3 and 7 days after transduction, fluorescence of cells was measured by flow cytometry (FACS) analysis. D) GFP expression into human iPS cells transduced with MS2RLPs. BBHX8 hiPSC cells were transduced with increasing amounts of MS2RLP-GFP, 105 to 106 pg of CAp24 per well, as indicated in the figure. 24 hours, after transduction, cells were harvested for FACS analysis. MFI: mean fluorescence intensity. FL2: red auto-fluorescence of the cells. E) Comparison of the efficacy of excision: Lane 1: Parental HCT116 cells; Lanes 2-6, non-transduced cells; Lane 3-7: cells transduced with a standard Lentiviral vector expressing the Cre recombinase. Lane 4-8: cells transfected with a Cre recombinase expression vector; Lane 5-9: Cells transduced with MS2RLP-Cre. F) GFP Knock-out using MS2RLP-Cas9N vs ILV-Cas9N. The monoclonal HCT116-GFP clone 1 copy cell line was first transduced by ILV-H1-Guide1-U6-Guide2 (both RNA guides targeting the GFP sequence) and then, transduced by MS2RLP-Cas9N or ILV-Cas9N. Two weeks after Cas9N transduction, the GFP knock out was analyzed by cytometry.

Supplementary Figures S3: A-B) MS2 tagging of HCV JFH1 SGR replicon. A) SGR-MS2-6X, SGR-MS2-12X and SGR-MS2-24X constructs were in vitro transcribed and transfected into Huh7.5 naïve cells. G418 treatment allowed selection of resistant clones, visualized by crystal violet staining. B) Functionality of transfected replicon was assessed by western blot detection of HCV non-structural proteins NS3 and NS5A. C) Efficient mobilization of MS2 tagged replicons by coat containing particles. Lentiviral particles were produced in MS2 tagged replicon harbouring Huh7.5, and filtered supernatants were used to transducer naïve Huh7.5 cells. After G418 treatment, neomycin resistant clones were fixed and crystal violet stained.

Supplementary Figures S4: Quantitative real time PCR analysis of A) RUNX2, B) DLX5 and C) PTHR1 expression in MG63 cells after RUNX2_MS2_12X or DLX5_MS2_12X mRNA transfer. MG63 cells were transduced with 50,000 PP or 100,000 PP per cell and cultured into αMEM medium with (A and B) or without BMP4 (C). 10 and 34 hours post-transduction, cells were harvested and the expression of RUNX2, DLX5 and PTHR1 was evaluated. D) Quantitative real time analysis of PTHR1 expression in MG63 cells 10 h and 34 h after osteoinduction with BMP4.

Supplementary Figures S5: A) Quantitative real time PCR analysis of endogenous RUNX2 and DLX5 expression in MSC after RUNX2_MS2_12X or DLX5_MS2_12X mRNA transfer (N=4). MSCs cells pre-cultured in EGM2 medium were transduced with 50,000 PP (MS2RLP-DLX5 or MS2RLP-RUNX2) per cell and maintained in αMEM supplemented with 2% FCS. 58h post-transduction, cells were harvested and the mRNA expression of endogenous RUNX2 and DLX5 were evaluated. B-C) Quantitative real time PCR analysis of STMN2 expression in MSC-l B) after RUNX2_MS2_12X mRNA transfer and C) into untransduced MSCs cells (N=4). MSCs cells pre-cultured in EGM2 medium were transduced with 50,000 PP (MS2RLP-DLX5 MS2RLP-RUNX2) per cell and maintained in αMEM supplemented with 2% FCS. 58h post-transduction, cells were harvested and the mRNA expression of STMN2 was evaluated. D) Quantitative real time PCR analysis of STMN2 expression in MSCs cells cultured with or without BMP4 and harvested 7 days after treatment.

Supplementary Figures S6: A) Up regulated genes according to stimulus conditions. B) Common upregulated genes between MS2RLP-DLX5 vs BMP4, MS2RLP-RUNX2 vs BMP4 and MS2RLP-RUNX2 vs MS2RLP-DLX5.

Supplementary Figures S7: A) Structure of the modified MLV vector. The MS2 sequence was introduced within the p12 sequence. B) Efficiency of replicon transfer. C) Image of G418 clones following MS2RLP transfer of HCV replicons.

Supplementary Table S1: Primers used for amplification. Sequence amplified for modifying the vectors (restriction sites used allowing the cloning are in bold).

Supplementary Table S2: List of upregulated and downregulated gene obtained after DLX5_MS2_12X mRNA transfer (Partek Software).

Supplementary Table S3: List of upregulated and downregulated gene obtained after RUNX2_MS2_12X mRNA transfer (Partek Software).

Supplementary Table S4: List of enriched pathway-based sets obtained after DLX5_MS2_12X mRNA transfer (http://cpdb.molgen.mpg.de/).

Supplementary Table S5: List of enriched pathway-based sets obtained after RUNX2-MS2_12X mRNA transfer (http://cpdb.molgen.mpg.de/).
