# Supplementary figures and images for: Highly efficient in vitro and in vivo delivery of functional RNAs using new versatile MS2-chimeric retrovirus-like particles
Source: Mol Ther Methods Clin Dev. 2015 Oct 21;2:15039–. doi: 10.1038/mtm.2015.39 (PMC4613645; doi:10.1038/mtm.2015.39)

## Slide 1
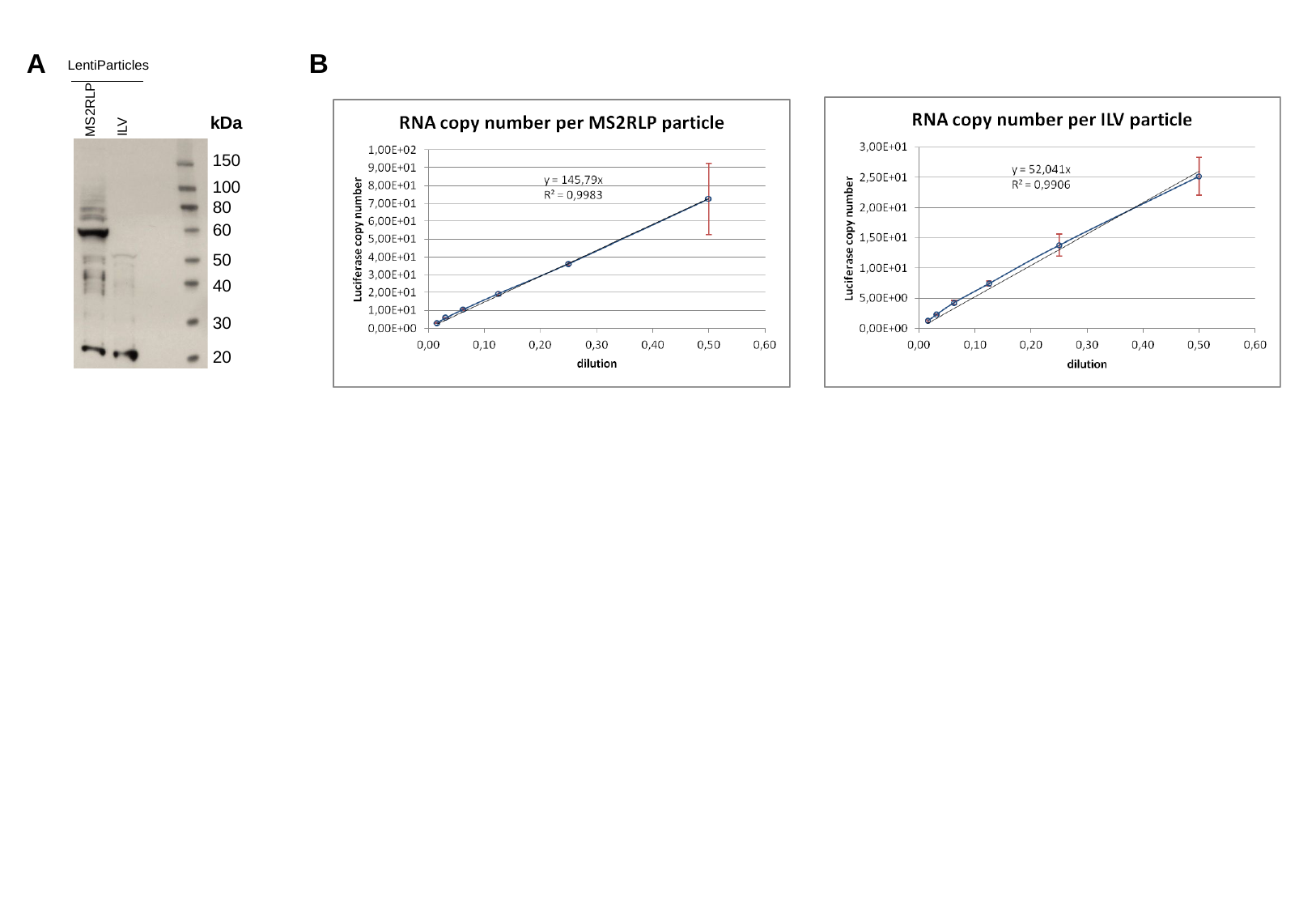

A
B
LentiParticles
MS2RLP
kDa
150
100
80
60
50
40
30
20
ILV

Supplement: Supplementary Figures S1: A) Analysis of GAG precursors maturation. 25 ng P24 from MS2RLP or ILV suspension were directed loaded onto the SDS-PAGE. The blot was probed with an anti-P24 antibody. B) Curves for the measurement of RNA copy number per MS2RLP particle. For this we collected supernatant f [file mtm201539-s1.pptx]

## Slide 1
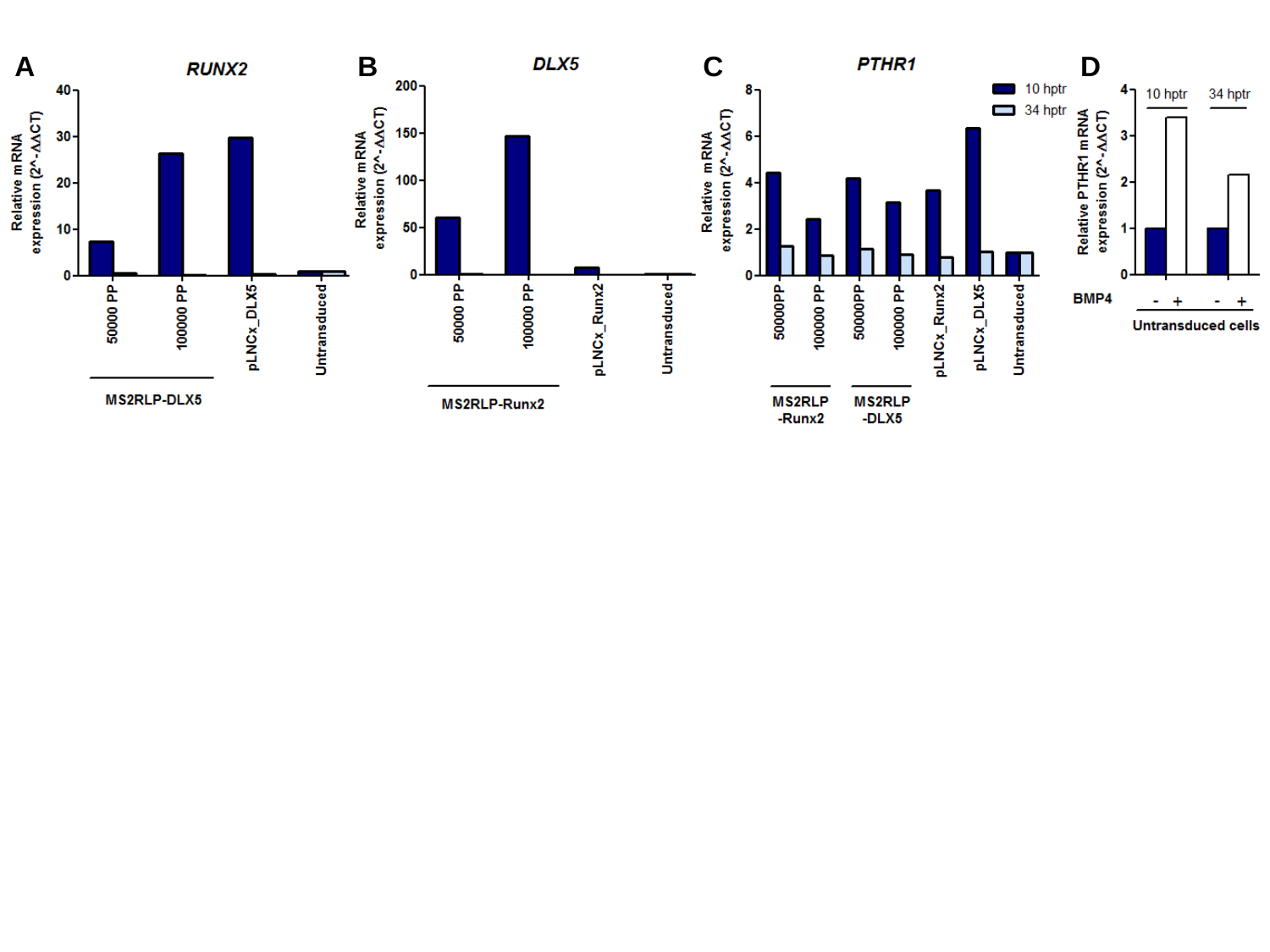

A
B
C
D

Supplement: Supplementary Figures S4: Quantitative real time PCR analysis of A) RUNX2, B) DLX5 and C) PTHR1 expression in MG63 cells after RUNX2_MS2_12X or DLX5_MS2_12X mRNA transfer. MG63 cells were transduced with 50,000 PP or 100,000 PP per cell and cultured into αMEM medium with (A and B) or without BMP4 (C [file mtm201539-s4.pptx]

## Slide 1
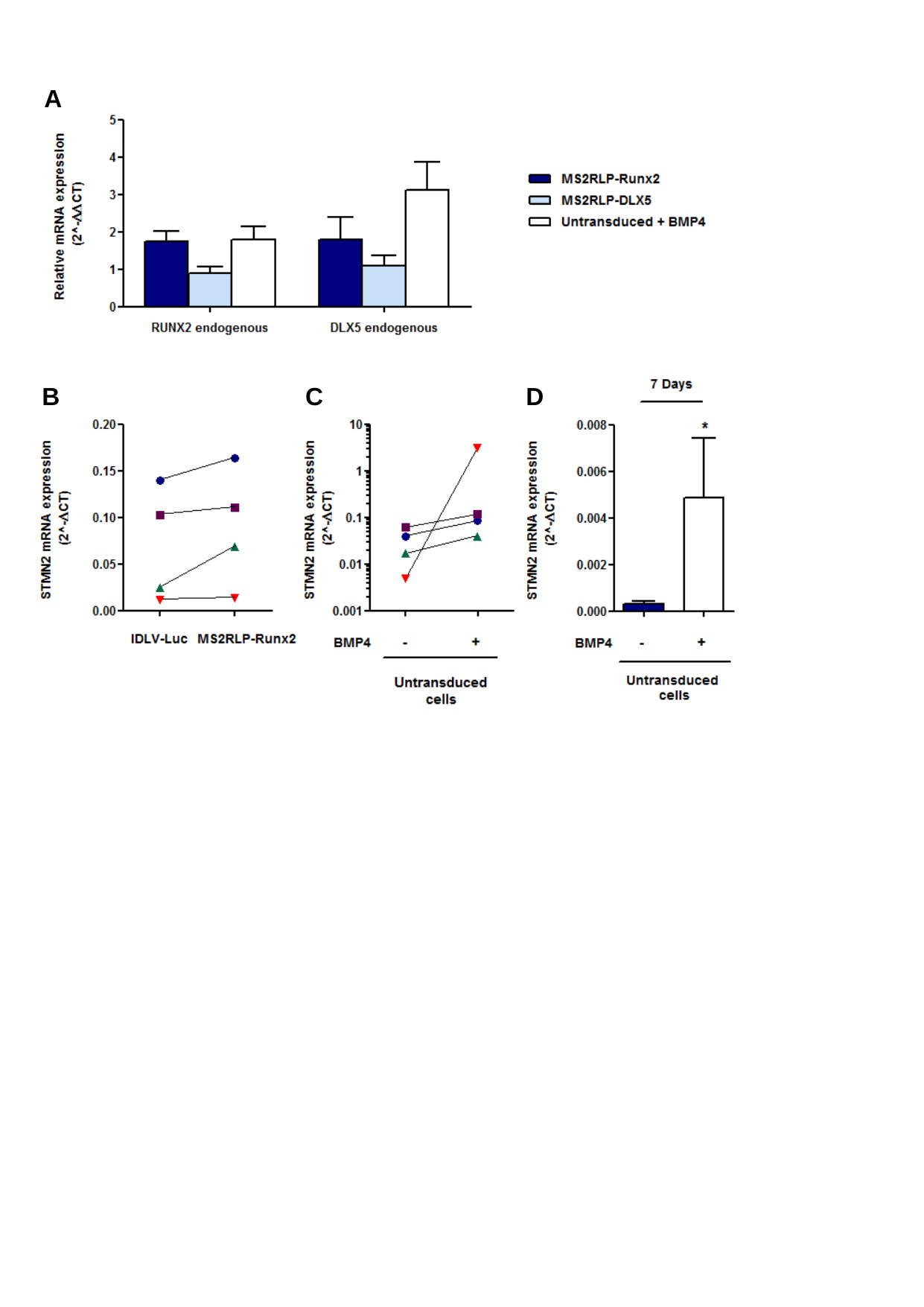

A
B
C
D

Supplement: Supplementary Figures S5: A) Quantitative real time PCR analysis of endogenous RUNX2 and DLX5 expression in MSC after RUNX2_MS2_12X or DLX5_MS2_12X mRNA transfer (N=4). MSCs cells pre-cultured in EGM2 medium were transduced with 50,000 PP (MS2RLP-DLX5 or MS2RLP-RUNX2) per cell and maintained in αMEM [file mtm201539-s5.pptx]
