# Supplementary material for: Highly efficient in vitro and in vivo delivery of functional RNAs using new versatile MS2-chimeric retrovirus-like particles
Source: Mol Ther Methods Clin Dev. 2015 Oct 21;2:15039–. doi: 10.1038/mtm.2015.39 (PMC4613645; doi:10.1038/mtm.2015.39)
Supplement: Supplementary Figures S2: A) Kinetics of luciferase activity upon MS2RLP transfer into HCT116 cells. HCT116 cells were transduced with MS2RLP-Luc (20 pg of Cap24/cell). At various times post-transduction (4H to 48H), cells were extensively washed, lysed, and luciferase activity was measured. B) GFP  [file mtm201539-s2.pptx]

## Slide 1
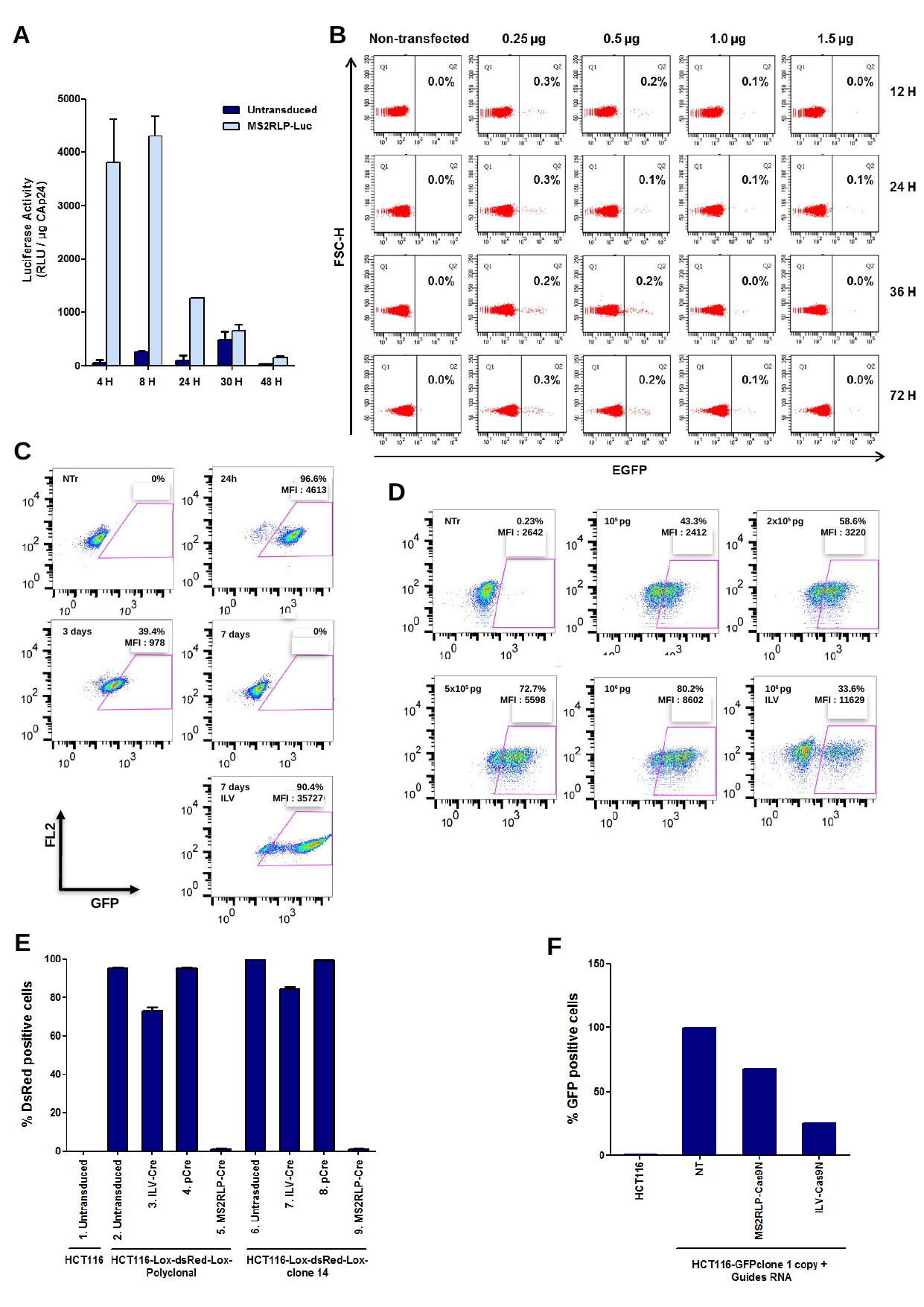

A
B
C
NTr
0%
24h
96.6%
MFI : 4613
3 days
39.4%
MFI : 978
0%
7 days
7 days
ILV
90.4%
MFI : 35727
FL2
GFP
D
NTr
0.23%
MFI : 2642
105 pg
43.3%
MFI : 2412
2x10^5^ pg
58.6%
MFI : 3220
5x10^5^ pg
72.7%
MFI : 5598
106 pg
80.2%
MFI : 8602
106 pg
ILV
33.6%
MFI : 11629
E
F
