# Supplementary material for: Highly efficient in vitro and in vivo delivery of functional RNAs using new versatile MS2-chimeric retrovirus-like particles
Source: Mol Ther Methods Clin Dev. 2015 Oct 21;2:15039–. doi: 10.1038/mtm.2015.39 (PMC4613645; doi:10.1038/mtm.2015.39)
Supplement: Supplementary Figures S3: A-B) MS2 tagging of HCV JFH1 SGR replicon. A) SGR-MS2-6X, SGR-MS2-12X and SGR-MS2-24X constructs were in vitro transcribed and transfected into Huh7.5 naïve cells. G418 treatment allowed selection of resistant clones, visualized by crystal violet staining. B) Functionality  [file mtm201539-s3.pptx]

## Slide 1
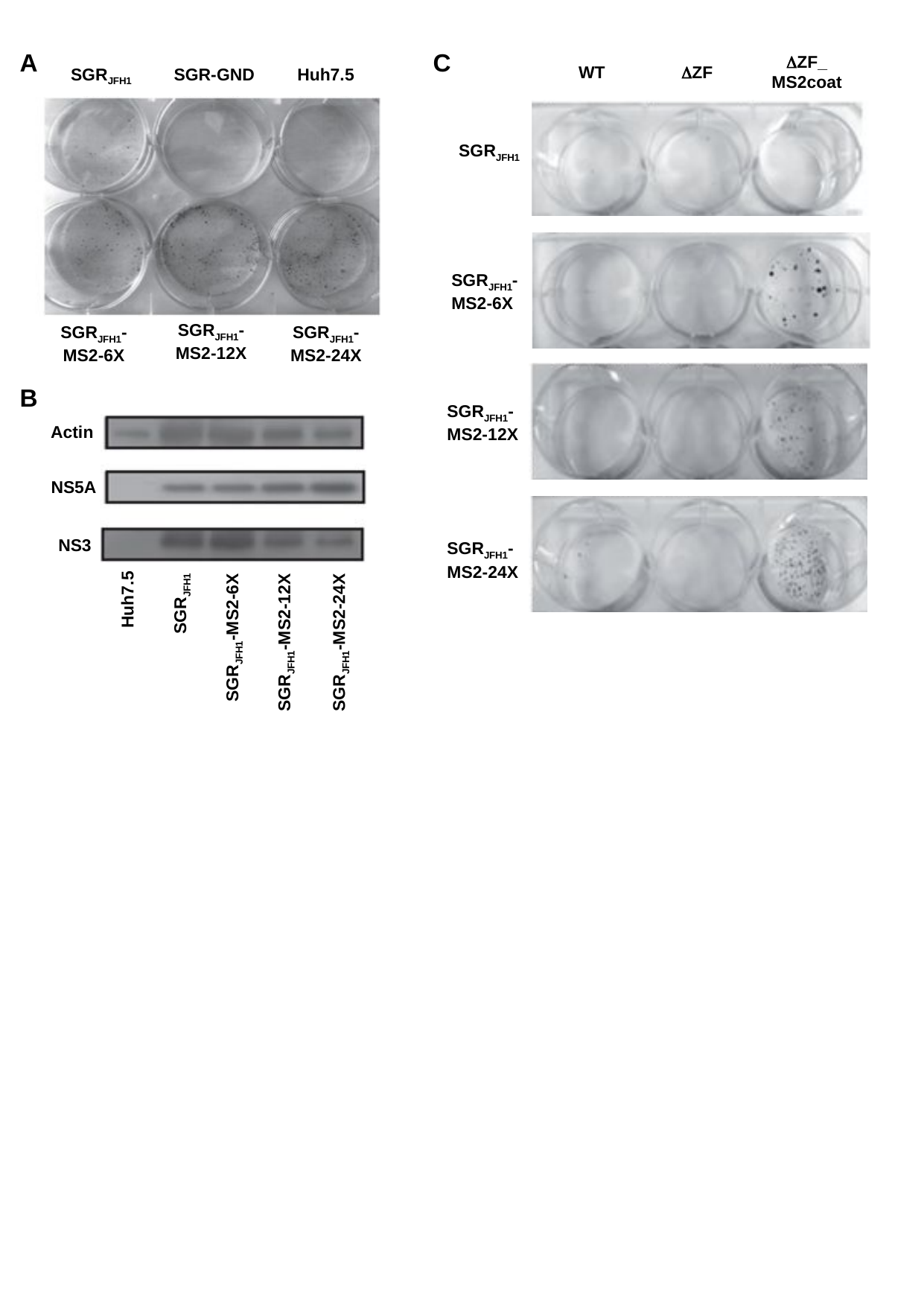

A
SGRJFH1
SGR-GND
Huh7.5
SGRJFH1-
MS2-12X
SGRJFH1-
MS2-24X
SGRJFH1-
MS2-6X
C
DZF_
MS2coat
WT
DZF
SGRJFH1
SGRJFH1-
MS2-6X
SGRJFH1-
MS2-12X
SGRJFH1-
MS2-24X
B
Actin
NS5A
NS3
Huh7.5
SGRJFH1
SGRJFH1-MS2-6X
SGRJFH1-MS2-12X
SGRJFH1-MS2-24X
