# Supplementary material for: Highly efficient in vitro and in vivo delivery of functional RNAs using new versatile MS2-chimeric retrovirus-like particles
Source: Mol Ther Methods Clin Dev. 2015 Oct 21;2:15039–. doi: 10.1038/mtm.2015.39 (PMC4613645; doi:10.1038/mtm.2015.39)
Supplement: Supplementary Figures S6: A) Up regulated genes according to stimulus conditions. B) Common upregulated genes between MS2RLP-DLX5 vs BMP4, MS2RLP-RUNX2 vs BMP4 and MS2RLP-RUNX2 vs MS2RLP-DLX5. [file mtm201539-s6.pptx]

## Slide 1
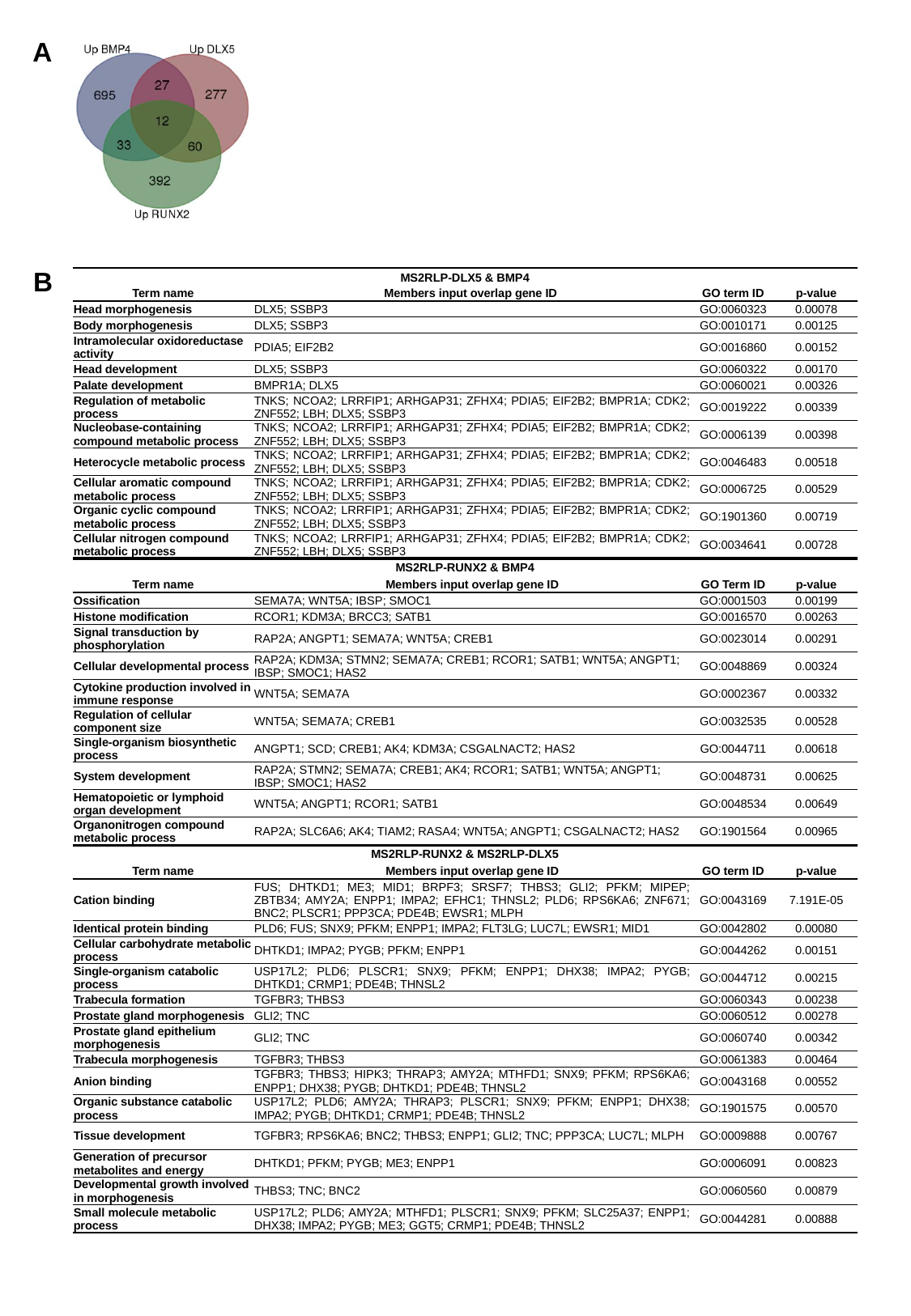

A
B
| MS2RLP-DLX5 & BMP4 | | | |
| --- | --- | --- | --- |
| Term name | Members input overlap gene ID | GO term ID | p-value |
| Head morphogenesis | DLX5; SSBP3 | GO:0060323 | 0.00078 |
| Body morphogenesis | DLX5; SSBP3 | GO:0010171 | 0.00125 |
| Intramolecular oxidoreductase activity | PDIA5; EIF2B2 | GO:0016860 | 0.00152 |
| Head development | DLX5; SSBP3 | GO:0060322 | 0.00170 |
| Palate development | BMPR1A; DLX5 | GO:0060021 | 0.00326 |
| Regulation of metabolic process | TNKS; NCOA2; LRRFIP1; ARHGAP31; ZFHX4; PDIA5; EIF2B2; BMPR1A; CDK2; ZNF552; LBH; DLX5; SSBP3 | GO:0019222 | 0.00339 |
| Nucleobase-containing compound metabolic process | TNKS; NCOA2; LRRFIP1; ARHGAP31; ZFHX4; PDIA5; EIF2B2; BMPR1A; CDK2; ZNF552; LBH; DLX5; SSBP3 | GO:0006139 | 0.00398 |
| Heterocycle metabolic process | TNKS; NCOA2; LRRFIP1; ARHGAP31; ZFHX4; PDIA5; EIF2B2; BMPR1A; CDK2; ZNF552; LBH; DLX5; SSBP3 | GO:0046483 | 0.00518 |
| Cellular aromatic compound metabolic process | TNKS; NCOA2; LRRFIP1; ARHGAP31; ZFHX4; PDIA5; EIF2B2; BMPR1A; CDK2; ZNF552; LBH; DLX5; SSBP3 | GO:0006725 | 0.00529 |
| Organic cyclic compound metabolic process | TNKS; NCOA2; LRRFIP1; ARHGAP31; ZFHX4; PDIA5; EIF2B2; BMPR1A; CDK2; ZNF552; LBH; DLX5; SSBP3 | GO:1901360 | 0.00719 |
| Cellular nitrogen compound metabolic process | TNKS; NCOA2; LRRFIP1; ARHGAP31; ZFHX4; PDIA5; EIF2B2; BMPR1A; CDK2; ZNF552; LBH; DLX5; SSBP3 | GO:0034641 | 0.00728 |
| MS2RLP-RUNX2 & BMP4 | | | |
| Term name | Members input overlap gene ID | GO Term ID | p-value |
| Ossification | SEMA7A; WNT5A; IBSP; SMOC1 | GO:0001503 | 0.00199 |
| Histone modification | RCOR1; KDM3A; BRCC3; SATB1 | GO:0016570 | 0.00263 |
| Signal transduction by phosphorylation | RAP2A; ANGPT1; SEMA7A; WNT5A; CREB1 | GO:0023014 | 0.00291 |
| Cellular developmental process | RAP2A; KDM3A; STMN2; SEMA7A; CREB1; RCOR1; SATB1; WNT5A; ANGPT1; IBSP; SMOC1; HAS2 | GO:0048869 | 0.00324 |
| Cytokine production involved in immune response | WNT5A; SEMA7A | GO:0002367 | 0.00332 |
| Regulation of cellular component size | WNT5A; SEMA7A; CREB1 | GO:0032535 | 0.00528 |
| Single-organism biosynthetic process | ANGPT1; SCD; CREB1; AK4; KDM3A; CSGALNACT2; HAS2 | GO:0044711 | 0.00618 |
| System development | RAP2A; STMN2; SEMA7A; CREB1; AK4; RCOR1; SATB1; WNT5A; ANGPT1; IBSP; SMOC1; HAS2 | GO:0048731 | 0.00625 |
| Hematopoietic or lymphoid organ development | WNT5A; ANGPT1; RCOR1; SATB1 | GO:0048534 | 0.00649 |
| Organonitrogen compound metabolic process | RAP2A; SLC6A6; AK4; TIAM2; RASA4; WNT5A; ANGPT1; CSGALNACT2; HAS2 | GO:1901564 | 0.00965 |
| MS2RLP-RUNX2 & MS2RLP-DLX5 | | | |
| Term name | Members input overlap gene ID | GO term ID | p-value |
| Cation binding | FUS; DHTKD1; ME3; MID1; BRPF3; SRSF7; THBS3; GLI2; PFKM; MIPEP; ZBTB34; AMY2A; ENPP1; IMPA2; EFHC1; THNSL2; PLD6; RPS6KA6; ZNF671; BNC2; PLSCR1; PPP3CA; PDE4B; EWSR1; MLPH | GO:0043169 | 7.191E-05 |
| Identical protein binding | PLD6; FUS; SNX9; PFKM; ENPP1; IMPA2; FLT3LG; LUC7L; EWSR1; MID1 | GO:0042802 | 0.00080 |
| Cellular carbohydrate metabolic process | DHTKD1; IMPA2; PYGB; PFKM; ENPP1 | GO:0044262 | 0.00151 |
| Single-organism catabolic process | USP17L2; PLD6; PLSCR1; SNX9; PFKM; ENPP1; DHX38; IMPA2; PYGB; DHTKD1; CRMP1; PDE4B; THNSL2 | GO:0044712 | 0.00215 |
| Trabecula formation | TGFBR3; THBS3 | GO:0060343 | 0.00238 |
| Prostate gland morphogenesis | GLI2; TNC | GO:0060512 | 0.00278 |
| Prostate gland epithelium morphogenesis | GLI2; TNC | GO:0060740 | 0.00342 |
| Trabecula morphogenesis | TGFBR3; THBS3 | GO:0061383 | 0.00464 |
| Anion binding | TGFBR3; THBS3; HIPK3; THRAP3; AMY2A; MTHFD1; SNX9; PFKM; RPS6KA6; ENPP1; DHX38; PYGB; DHTKD1; PDE4B; THNSL2 | GO:0043168 | 0.00552 |
| Organic substance catabolic process | USP17L2; PLD6; AMY2A; THRAP3; PLSCR1; SNX9; PFKM; ENPP1; DHX38; IMPA2; PYGB; DHTKD1; CRMP1; PDE4B; THNSL2 | GO:1901575 | 0.00570 |
| Tissue development | TGFBR3; RPS6KA6; BNC2; THBS3; ENPP1; GLI2; TNC; PPP3CA; LUC7L; MLPH | GO:0009888 | 0.00767 |
| Generation of precursor metabolites and energy | DHTKD1; PFKM; PYGB; ME3; ENPP1 | GO:0006091 | 0.00823 |
| Developmental growth involved in morphogenesis | THBS3; TNC; BNC2 | GO:0060560 | 0.00879 |
| Small molecule metabolic process | USP17L2; PLD6; AMY2A; MTHFD1; PLSCR1; SNX9; PFKM; SLC25A37; ENPP1; DHX38; IMPA2; PYGB; ME3; GGT5; CRMP1; PDE4B; THNSL2 | GO:0044281 | 0.00888 |
