# Supplementary material for: Highly efficient in vitro and in vivo delivery of functional RNAs using new versatile MS2-chimeric retrovirus-like particles
Source: Mol Ther Methods Clin Dev. 2015 Oct 21;2:15039–. doi: 10.1038/mtm.2015.39 (PMC4613645; doi:10.1038/mtm.2015.39)
Supplement: Supplementary Figures S7: A) Structure of the modified MLV vector. The MS2 sequence was introduced within the p12 sequence. B) Efficiency of replicon transfer. C) Image of G418 clones following MS2RLP transfer of HCV replicons. [file mtm201539-s7.pptx]

## Slide 1
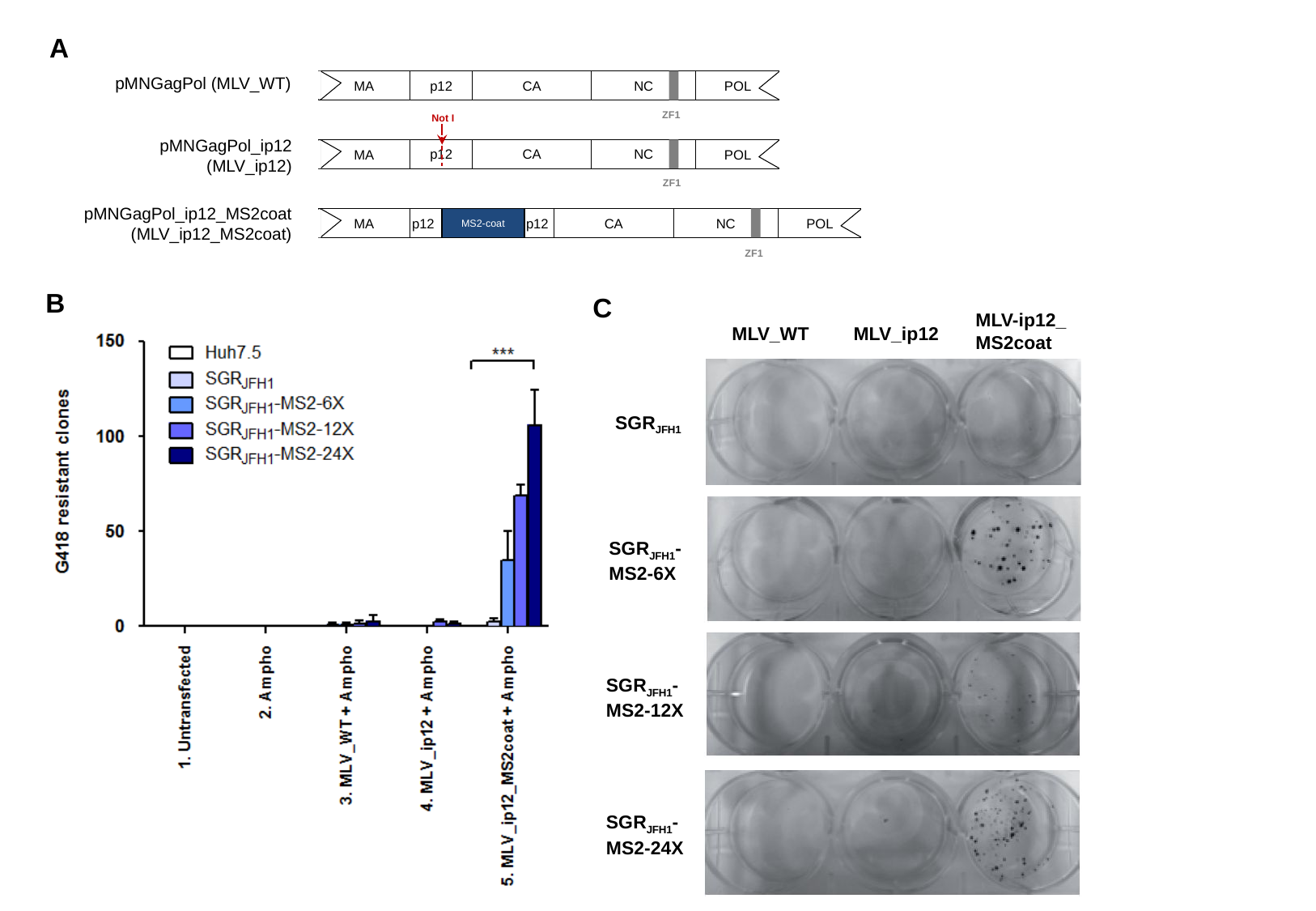

A
pMNGagPol (MLV_WT)
MA
p12
CA
NC
POL
ZF1
Not I
pMNGagPol_ip12
 (MLV_ip12)
p12
CA
NC
MA
POL
ZF1
pMNGagPol_ip12_MS2coat
 (MLV_ip12_MS2coat)
p12
p12
CA
NC
MA
POL
MS2-coat
ZF1
B
C
MLV-ip12_
MS2coat
MLV_WT
MLV_ip12
SGRJFH1
SGRJFH1-
MS2-6X
SGRJFH1-
MS2-12X
SGRJFH1-
MS2-24X
