# Supplementary material for: Highly efficient in vitro and in vivo delivery of functional RNAs using new versatile MS2-chimeric retrovirus-like particles
Source: Mol Ther Methods Clin Dev. 2015 Oct 21;2:15039–. doi: 10.1038/mtm.2015.39 (PMC4613645; doi:10.1038/mtm.2015.39)
Supplement: Supplementary Table S1: Primers used for amplification. Sequence amplified for modifying the vectors (restriction sites used allowing the cloning are in bold). [file mtm201539-s8.docx]

| **Target** | **Primer** | **Primer Sequence (5'-3')** |
| --- | --- | --- |
| p8.74 | p8.74bZF-for | CC**gCggCCgC**gTTgACgCgCA |
|  | p8.74bZF-rev | CAgT**gTTAAC**gCCCTTTTTCCTAgg |
|  | p8.74aZF-for | AT**gCggCCgC**gTTAACACTgAgAgACAgg |
|  | p8.74aZF-rev | ggCT**gATATC**TAATCCCTggTgTCTC |
| MS2 Coat | MS2Coat-HpaI-for | TTgTTAACATggCTTCTAACTTT |
|  | MS2Coat-HpaI-rev | CCgTTAACgTAgATgCCggAgTT |
| MS2 Stem loops | MS2-BamHI-for | ACCCgggCCCTATATAT**ggATCC** |
|  | MS2-BamHI-6X-rev | AT**ggATCC**gTgATTCCCCg |
|  | MS2-BamHI-12X-rev | TAggCAATTA**ggATCC**TTAggAT |
| Luciferase  (coding sequence) | Luciferase-S | TCg**AAgCTT**ACCATggAAgACCCA |
|  | Luciferase-AS | gAAT**ggTACC**TTACAATTTggAC |
| JFH1 | JFH1-for | TCTgCggAACCggTgAgTA |
|  | JFH1-rev | TCAggCAgTACCACAAggC |
| beta - Actin | Actin-for | CgCACCACTggCATTgTCAT |
|  | Actin-rev | TTCTCCTTgATgTCACgCAC |
| Luciferase | Luciferase-qPCR-for | CAACTgCATAAggCTATgAAgAgA |
|  | Luciferase-qPCR-rev | ATTTgTATTCAgCCCATATCgTTT |
| RUNX2 | Runx2-for | CACTggCgCTgCAACAAgAC |
|  | Runx2-rev | ggCCCACAAATCTCAgATCgTT |
| DLX5 | DLX5-for | gCCACCAACCAgCCAgAgAA |
|  | DLX5-rev | gCgAggTACTgAgTCTTCTgAAACC |
| PTHR1 | PTHR1-for | ACATCTgCgTCCACATCAgg |
|  | PTHR1-rev | CCgTTCACgAgTCTCATTggTg |
| iBSP | iBSP-for | gggCAgTAgTgACTCATCCgAAg |
|  | iBSP-rev | CTCCATAgCCCAgTgTTgTAgCAg |
| OSTERIX | Osterix-for | CTCCTgCgACTgCCCTAAT |
|  | Osterix-rev | gCCTTgCCATACACCTTgC |
| RUNX2 endogenous | RUNX2-UTR-for | TCCCTCTgAAAAggCAgCAgg |
|  | RUNX2-UTR-rev | gCATgCCACAgAAggACTCT |
| DLX5 endogenous | DLX5-UTR-for | TggggAAgTTCggAAAACTgA |
|  | DLX5-UTR-rev | AggCACCATTgAAAgTgTCCA |
| STMN2 | STMN2-for | gCggAggAAAAgCTgATCCTgA |
|  | STMN2-rev | TCCgCAgCATgCCTCTCCTT |
| GAPDH | GAPDH-for | CTggCgCTgAgTACgTCg |
|  | GAPDH-rev | TTgACAAAgTggTCgTTgA |
| PPIA | PPIA-for | gCCgAggAAAACCgTgTACTAT |
|  | PPIA-rev | TCTTTgggACCTTgTCTgCAA |
