# Supplementary material for: Highly efficient in vitro and in vivo delivery of functional RNAs using new versatile MS2-chimeric retrovirus-like particles
Source: Mol Ther Methods Clin Dev. 2015 Oct 21;2:15039–. doi: 10.1038/mtm.2015.39 (PMC4613645; doi:10.1038/mtm.2015.39)
Supplement: Supplementary Files [file mtm201539-s13.doc]

Supplemental material and methods

***Cell line and culture condition***

The MG63 cell (derived from the clone ATCC CRL-1427) were cultured in Minimum Essentiel medium alpha (αMEM) (Glutamax) supplemented with penicillin (100U/mL), streptomycin (100 µg/mL) and 10% heat inactivated fetal calf serum (FCS).

***MLV derived retroviral vectors***

MS2 Coat protein was inserted into p12 coding sequence of pMNGagPol retroviral vector using PCR added NotI restriction site. NotI restriction site was inserted in p12 using assembly PCR. Upstream region of insertion site was amplified using GagPolAflII-for: CTAgCTAGCCTTAAgTTTgACCTTAgg AflII restriction site containing primer and GagPolp12-NotI-rev: TTAgCggCCgCgCCAggAgTgAgggCT NotI restriction site containing primer. Downstream region of insertion site was amplified using GagPolp12-NotI-for: gAATgCggCCgCgTCTCTAgg, containing NotI restriction site and GagPolp12-AflII-rev: ATTCTTAAgTCTCTCTAggAAggCCgA containing AflII restriction site. After sub cloning into pGEMT-easy vector (Promega), pMNGagPolp12NotI (MLV_ip12) was generated by AflII/NotI cloning. MS2 Coat coding sequence was amplified from LMNI-MS2-NLS-GFP plasmid, kind gift of P.D. Bieniasz, using primers containing NotI restriction sites MS2Coat-NotI-for: ATgCggCCgCAATggCTTCTAACTT and MS2Coat-NotI-rev : gTTTAgCggCCgCgTAgATgC and was inserted into MLV_ip12 by NotI in-frame cloning, generating pMNGagPol_ip12-MS2-Coat (MLV_ip12_MS2coat) plasmid.

***Western blotting***

To analysis GAG precursor maturation, 25ng P24 from lentiparticles were loaded on 4-12% SDS-PAGE gels. The gels were transferred onto a PVDF membrane using a Trans-Blot SD apparatus (Bio-Rad). The membrane was incubated with anti-P24 [clone 39/5.4A, Abcam). Western blot was revealed using the **Pierce™ Fast Western Blot Kit, ECL Substrate.** Visualization of the protein bands was achieved by the chemiluminescence method, and the films were developed and fixed.

**GFP expression in Hela cells**

50x10^3^ Hela cells were seeded in 6 well-plates 24 h before transduction. Approximately 1x10^8^ physical particles per well were used. After 24 h, cells were washed with saline solution and analysed by flow cytometry (FACS) or cultured for a total of additional seven days to follow GFP expression.

**Transduction of human induced pluripotent stem cells**

The BBHX8 hiPSC line was maintained on Matrigel-coated culture wells (BD Biosciences, San Jose, CA, USA) in mTeSR1 (Stemcell Technologies, Vancouver, BC, Canada) at 37 °C in 5% CO_2_ incubator with daily medium change and by using Gentle Cell Dissociation Reagent (Stemcell Technologies, Vancouver, BC, Canada) for cell passaging. 3 days before transduction, 200x10^3^ cells were seeded in 12-wells plate. Transduction was performed for 24 h with different amount of viral particles, in ng of CAp24, and with cells at 60-70% confluence. Before FACS analysis cells were detached using TrypLE (Life Technologies, Carlsbad, CA, USA).

**CRISPR experiment**

HCT116-GFP clone 1 copy were obtained by transduction of HCT116 by a GFP-expressing lentiviral vector. The monoclonal HCT116-GFP clone 1 copy cell line was selected by limit dilution of the polyclonal cell line and screened by qPCR based on the GFP integrated copy number. The monoclonal cell line was transduced by an ILV lentiviral vector carrying a couple of guides sequences targeting the GFP sequence. Then, the HCT116-GFP clone 1 copy transduced by the ILV-Guides were transduced by MS2RLP-Cas9N or ILV-Cas9N in presence of 8µg/mL Polybrene. Two weeks after transduction, cells were trypsinized and analyzed by cytometry (Mylteniy MACS Quant VYB).

***MG63 transduction with lentiviral particles***

To evaluate the differentiation of MG63 cells after DLX5-MS2_12X or RUNX2-MS2_12X mRNA, cells (300,000 cells/ well in 24 well-plate) were transduced with 50x10^3^ or 100x10^3^ viral particles per cell and maintained in αMEM supplemented with 2% FCS, ascorbic acid (50µM), β-glycerophosphate (10mM), penicillin (100U/mL) and streptomycin (100 µg/mL) or αMEM supplemented with 2% FCS, ascorbic acid (50 µM), β-glycerophosphate (10 mM), BMP4 (50 ng/mL), penicillin (100 U/mL) and streptomycin (100 µg/mL). Transduction was improved by the addition of polybrene® (5 µg/mL). After 10h, medium was removed and replaced by fresh medium with or without BMP4. 30h after the first transduction, cells were transduced again as above. At 10 h and 34 h, cells were washed 3 times with PBS and harvested. Then, total RNA was extracted using RNeasy microkit (Qiagen).

**Quantitative real-time PCR**

First strand cDNA was prepared using the High capacity reverse transcription kit (Applied biosystems) with 100 ng extracted total RNA and random primers. The expression of osteoblastic transcription factor *RUNX2*, *DLX5* and *PTHR1* was evaluated by real-time quantitative PCR using EvaGreen incorporation (SsoFast EvaGreen kit, CFX96 Real-time PCR detection system, Biorad). PCR amplification of the reaction mixture (5 µl of reverse transcriptase product diluted at 10:1 in RNAse-, DNAse-free water, 1X SsoFast EvaGreen kit, 10µM each primer (table1) and RNase –free water to a final volume of 20µl) involved 3 min pre-amplification at 95°C, 40 cycles of 10’’ at 95°C and 30’’ at 60°C, then melting curve and cooling steps. The GAPDH gene was used as a normalization control.
